# Examining the role of wind in human illness due to pesticide drift in Washington state, 2000–2015

**DOI:** 10.1186/s12940-021-00693-3

**Published:** 2021-03-15

**Authors:** Edward J. Kasner, Joanne B. Prado, Michael G. Yost, Richard A. Fenske

**Affiliations:** 1 Department of Environmental and Occupational Health Sciences, University of Washington School of Public Health, Seattle, WA USA; 2 Washington State Department of Health, Tumwater, WA USA

**Keywords:** Pesticide spraying, Application exclusion zone, Drift, Acute pesticide-related illness, Meteorology, Wind ramping

## Abstract

**Background:**

Pesticides play an important role in protecting the food supply and the public’s health from pests and diseases. By their nature, pesticides can be toxic to unintended target organisms. Changing winds contribute to pesticide drift— the off-target movement of pesticides—and can result in occupational and bystander illness.

**Methods:**

We systematically linked historical weather data to documented pesticide drift illnesses. We used Washington State Department of Health data to identify 252 drift events that included 690 confirmed cases of illness from 2000 to 2015. To characterize wind speed and direction at the time of the events, we paired these data with meteorological data from a network of 171 state weather stations. We report descriptive statistics and the spatio-temporal extent of drift events and compare applicator-reported weather conditions to those from nearby meteorological stations.

**Results:**

Most drift events occurred in tree fruit (151/252 = 60%). Ground spraying and aerial applications accounted for 68% and 23% of events, respectively; 69% of confirmed cases were workers, and 31% were bystanders. Confirmed cases were highest in 2014 (129) from 22 events. Complete applicator spray records were available for 57 drift events (23%). Average applicator-reported wind speeds were about 0.9 m •sec^− 1^ (2 mi •hr^− 1^) lower than corresponding speeds from the nearest weather station values.

**Conclusions:**

Drift events result from a complex array of factors in the agricultural setting. We used known spatio-temporal aspects of drift and historical weather data to characterize these events, but additional research is needed to put our findings into practice. Particularly critical for this analysis is more accurate and complete information about location, time, wind speed, and wind direction. Our findings can be incorporated into new training materials to improve the practice of pesticide application and for better documentation of spray drift events. A precision agriculture approach offers technological solutions that simplify the task of tracking pesticide spraying and weather conditions. Public health investigators will benefit from improved meteorological data and accurate application records. Growers, applicators, and surrounding communities will also benefit from the explanatory and predictive potential of wind ramping studies.

**Supplementary Information:**

The online version contains supplementary material available at 10.1186/s12940-021-00693-3.

## Background

Pesticides play an important role in protecting the food supply and the public’s health from pests and diseases [1, 2]. By their nature, pesticides can be toxic to unintended target organisms [3]. Agencies track the impacts of pesticide use to ensure that they don’t pose unreasonable burdens. For example, the Washington State Departments of Health (WADOH), Agriculture (WSDA), and Labor and Industries (L&I) cooperatively implement the federal Agricultural Worker Protection Standard [4]. WADOH coordinates a public health surveillance program that uses a standardized case definition to document pesticide-related illness and develops strategies to prevent human exposure [5–8].

Under Washington State law (RCW 70.104.030), WADOH is authorized to secure the information that is necessary to adequately determine the nature and cause of pesticide illness cases. All licensed pesticide applicators (agricultural and non-agricultural) and anyone applying pesticides to more than one acre of agricultural land per calendar year are required to keep application records (i.e., spray records). Additionally, regardless of applicator license status, public entities must keep spray records—for roadside applications, as must anyone making landscape applications to commercial properties, parks, schools, and other public places. As it relates to wind, the state requires an applicator to record direction and estimated velocity of the wind during the time the pesticide was applied, but it does not specify a standardized method for measuring these variables. Common practice described in training is for an applicator to take a measurement with a handheld anemometer on the upwind side of a sprayed area outside the tree canopy.

The off-target movement of pesticides, or drift, from application sources to human receptors represents an important exposure pathway in agricultural areas [9]. Nationally, drift accounts for 37–54% of pesticide-related illnesses among agricultural workers in the United States [10, 11]. Compared to bystander drift exposure, illness from occupational drift exposure tends to be reported with similar frequency but higher severity [9]. We define a bystander as an individual near an area being sprayed who is engaged in an activity that is neither work-related nor involved with the spray application itself [12]. Drift remains a public health concern in the Pacific Northwest. In 2010–2011, WADOH estimated that 51% (67/131) of cases resulting from agricultural applications were drift-related and that 64% (43/67) of those were workers drifted on from an adjacent farm [4]. In May 2014, WADOH reported that 60 individuals, mostly orchard workers, became ill in 15 drift events over a two-month period—a number normally seen over an entire year [13, 14]. Of particular focus in Washington State are drift events associated with axial fan sprayers, also known as airblast sprayers [15–18]. This application equipment is used on multiple crops, including tree fruit, grapes, and hops. These concerns led to the creation of a Pesticide Application Safety Committee by the Washington State Legislature in 2019 [19].

Unfavorable wind is a leading contributing factor for illnesses resulting from pesticide drift [9, 20]. However, the effects of changing wind speed and direction on human exposure during a pesticide application aren’t well understood. Computer modeling shows that uncontrollable meteorological variables such as wind speed and direction can change rapidly [21]. Meteorological conditions are an important component of the environmental fate and transport of spray droplets. Transport is also influenced by the droplet size and release height of the spray [22, 23]. Smaller droplets increase crop coverage, but they are drift-prone due to weaker gravitational forces and greater wind speed and direction changes. These influencing factors are also an indicator of atmospheric turbulence and droplet dilution [21, 24]. The agriculture and public health sectors can make good use of meteorological data to understand, forecast, and reduce exposure to pesticide drift.

Regulatory agencies differentiate primary spray drift from secondary off-target movement. Primary spray drift occurs during an application or soon thereafter. Secondary, off-target movement occurs well after application by means of volatilization or resuspension on dust particles. The Spray Drift Task Force (SDTF), formed by pesticide registrants in collaboration with the United States Environmental Protection Agency (USEPA), defines spray drift as the movement of droplets during or soon after application and irrespective of pesticide active ingredient. Contributing factors include environmental and meteorological conditions, spray technique, and crop type [25, 26]. This paper refers to spray drift as *drift*.

To understand the mechanisms of pesticide drift exposure, new approaches are needed. Several studies have incorporated weather information while monitoring pesticide drift, but they weren’t tied to cases of pesticide drift illnesses [27, 28]. The Washington State University (WSU) AgWeatherNet (AWN) system provides remote, real-time weather monitoring on its website. This data assists growers with customized weather alerts and decision support systems to “help improve production and product quality, optimize resource use, and reduce environmental impact” [29, 30]. This study explores the use of meteorological data in the context of public health practice in Washington State. We linked data from WADOH and AWN to characterize wind speed and direction during agricultural drift events that involved individuals between the years 2000 and 2015. To lay the groundwork for predicting and preventing future drift events, we worked to gain a deeper understanding of meteorological conditions for epidemiological investigations of pesticide illness.

We determined the spatial and temporal aspects of drift events in Washington State over a 16-year period and developed best estimates of wind speed and direction near the time of exposure. We characterized regions that are susceptible to drift events by summarizing target crops, method of application, work activity of individuals at the time of exposure, and number of individuals who reported a drift-related illness for each event.

## Methods

We reviewed records from the WADOH pesticide illness database for agricultural drift events that occurred between the years 2000 and 2015. We developed a geographic information system (GIS) to link geocoded and time-specific drift event data to agricultural land use and weather geospatial layers. The Washington State Institutional Review Board determined that the project was research not involving human subjects.

### Pesticide illness database

Each year, WADOH investigates hundreds of pesticide illness reports to determine whether there is a causal relationship between adverse health signs or symptoms and pesticide exposure [4]. To make a determination, WADOH investigators conduct interviews; take field visits; and review pesticide spray records, medical records, and reports from other state agencies [4]. They use a standardized case classification system that categorizes illnesses as *Definite*, *Probable*, *Possible*, *Suspicious*, *Unlikely*, *Insufficient information*, *Asymptomatic*, or *Unrelated* [7, 31]. In this study, we restricted the analysis to *Definite*, *Probable*, and *Possible* cases and refer to them collectively as “confirmed” cases that are based on a preponderance of evidence. Confirmed cases had documentation of: (1) a pesticide exposure pathway, (2) adverse health effects (symptoms and/or signs of illness), and (3) toxicological evidence supporting a causal relationship between the observed pesticide exposure and the resulting health effects in a likely time frame [4]. We classified all other reports associated with drift events as “unconfirmed” cases.

Data in this study were limited to confirmed cases resulting from pesticide applications that drifted in agricultural settings. We defined a *drift case* as an individual who reported adverse health effects after being exposed via the movement of pesticide spray, mist, fumes, or odor that was carried away from the treatment site target by air [7]. A *drift event* was as an incident where one or more drift cases experienced drift exposure from a single source [9] (Supplementary Fig. S1). We determined *drift event size* by counting the number of confirmed cases associated with each drift event. Our analysis included the following variables from the WADOH database: date and time of exposure, activity of individual at the time of exposure, work-relatedness of the exposure, and illness severity category. We gathered additional spatio-temporal and weather data from state investigation reports (WSDA and L&I) and spray records that were kept by pesticide applicators. State investigation reports provided location and distance of exposed individual(s) from the spray source. We also extracted date and time of application, equipment used for application, target crop being sprayed, applicator-reported wind speed and direction, and location from spray records.

### Drift event geocoding

All drift event locations were processed according to their estimated latitude and longitude (lat/long) geocoordinates, as determined by the following hierarchical workflow: if an event didn’t have lat/long, then the centroid (geometric center) of the one square mile Township/Range/Section (TRS) was considered the next optimal choice for rural areas, followed by street address, city centroid, and zip code centroid. We performed geocoding using Washington Master Addressing Services, a Microsoft Excel add-in tool [32–34]. We converted each drift event location to lat/long geocoordinates for displaying as a GIS layer. To ensure accuracy, we checked approximately 10% of randomly selected geocoded locations against land use and satellite data imagery.

### Agricultural land use data layer

We downloaded an agricultural land use geodatabase that was created by WSDA. The geodatabase contains annual estimates of crop area and type in a regularized grid of one square mile (640 acre, 259 ha) sections [35, 36]. These sections match the naming structure of the Township/Range/Section feature class that was originally developed by the Public Land Survey System (PLSS) [37]. The PLSS divides land into six-mile square townships. Each township is subdivided into 36 one-mile square sections. Each section is identified by a unique Township (north-south) and Range (east-west) designation [37].

### Meteorological data layer

We extracted geocoordinates and historical wind data from a database of 57 million measurements taken by 171 AWN meteorological stations throughout Washington State between the years 2000 and 2015 [29, 38]. Standardized sensors on each AWN station measured air temperature and relative humidity, soil temperature and moisture, wind speed and direction, leaf wetness, rainfall, solar radiation, and air pressure [39]. Meteorological data are collected with a 0.2 Hz sampling frequency by a data logger (*Campbell Scientific CR-1000;* Logan, UT), processed as 15-min averages, and viewable through an online portal. Variables of primary interest included average wind speed and direction (reported in degrees, both continuous and categorized into 16 principal directions of 22.5° increments). Wind gust data were not available for this analysis, which included some drift events that happened in a matter of minutes.

### Spatio-temporal relationships

We developed a geographic information system (GIS) in ArcMap 10.3 to link the drift event, land use, and weather network data layers [40] following technical standards [41, 42]:

- North American Datum of 1983 and High Accuracy Reference Network
- Lambert Conic Conformal projection system
- Washington State Plane Coordinates system (South Zone)
- Lat/long converted to coordinate units of US Survey Feet with ±40 ft accuracy

**Fig. 1:**
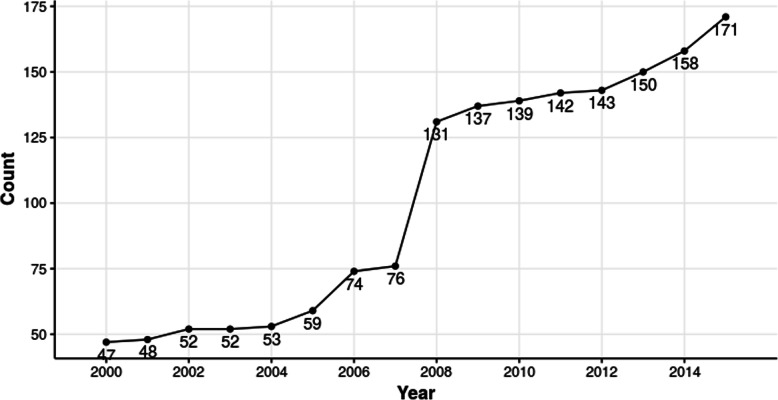
Number of AgWeatherNet (AWN) weather stations available by year, 2000–2015

To generate best estimates of wind speed and direction for the time and location of each drift event, we drew on historical data from AWN using a nearest-neighbor approach. We used the “Generate Near Table” proximity tool in ArcMap 10.3 to find the point-to-point Euclidean planar distance between each drift event and the 10 nearest AWN stations as of 2015 [43]. We anticipated that distance to the nearest station would be substantially smaller for the second half of the study period because of a near-doubling of network stations between 2007 and 2008 (Fig. 1).

### Applicator-reported and weather station conditions during sprays

With respect to wind speed, applicators recorded two discrete values on spray records: minimum and maximum. We used the arithmetic mean of these two values to find average applicator-reported wind speed (AR-Avg). AWN wind speed was measured every 5 s, averaged to 15-min intervals, and then logged. For all drift events involving complete spray records, we used scatterplots to examine the association between applicator self-reported wind speed (e.g., 5 mph average taken from 3 to 7 mph range on spray record) and AWN wind speed (e.g., 5 mph average from spray start time or the entire spray period). The line of unity in these scatterplots represents perfect agreement between the two values, which were evaluated based on the two different timeframes: spray start time only and entire time elapsed between spray start and stop times (i.e., spray period). We computed the difference between applicator self-reported wind speed and AWN wind speed for the application start time (hh:mm). We repeated this calculation by substituting AWN wind speed for the entire spray period (e.g., 4-h average). Under the assumption that the differences followed an approximately normal distribution, we used two-sided paired t-tests to evaluate the null hypothesis that the true mean difference between applicator-recorded and AWN wind speeds was zero at the α = 0.05 level. We reported point estimates, 95% confidence intervals, and *p*-values.

### Analysis

We worked with findings that were reported for all crops and, in many cases, for tree fruit only, given the high frequency of drift events associated with airblast spraying. We managed and analyzed data with R version 3.4.0 (2017-04-21) using the following packages: circular, bookdown, ggplot2, ggthemes, gridExtra, knitr, lubridate, and reshape. We produced descriptive statistics tables; scatter, bar, and time series plots; and choropleth maps.

## Results

Between the years 2000 and 2015, we identified 252 drift events involving 738 individuals, 690 of whom were confirmed cases (Table 1). When restricted to tree fruit only, there were 151 drift events involving 320 confirmed cases (Table 2).

**Table 1:** Drift event size by case status, all crops, 2000–2015

Event size^a^	Events		Confirmed cases		Unconfirmed cases	
	n	(%)	n	(%)	n	(%)
1	162	(64.3)	162	(23.5)	9	(18.8)
2	39	(15.5)	78	(11.3)	3	(6.3)
3	16	(6.3)	48	(7.0)	4	(8.3)
4	12	(4.8)	48	(7.0)	–	–
5	3	(1.2)	15	(2.2)	7	(14.6)
6+	20	(7.9)	339	(49.1)	25	(52.1)
Total	252	(100)	690	(100)	48	(100)

a. Event size reflects the number of cases involved in a drift event

**Table 2:** Intended application target in drift events by case status, all crops, 2000–2015

Application target	Events		Confirmed cases		Unconfirmed cases	
	n	(%)	n	(%)	n	(%)
Tree fruit	151	(59.9)	320	(46.4)	30	(62.5)
Undesired plant	23	(9.1)	41	(5.9)	–	–
Vegetable	21	(8.3)	124	(18.0)	8	(3.2)
Soil	12	(4.8)	66	(9.6)	–	–
Cereal	12	(4.8)	30	(4.3)	–	–
Small fruit	8	(3.2)	12	(1.7)	–	–
Other grain/fiber	6	(2.4)	22	(3.2)	3	(6.3)
Grass	3	(1.2)	49	(7.1)	–	–
Beverage crop	3	(1.2)	7	(1.0)	7	(14.6)
Landscape/ornamental	3	(1.2)	6	(0.9)	–	–
Oil crop	3	(1.2)	5	(0.7)	–	–
Flavoring/spice	3	(1.2)	4	(0.6)	–	–
Forest	2	(0.8)	2	(0.3)	–	–
Building surface	1	(0.4)	1	(0.1)	–	–
Other fruit	1	(0.4)	1	(0.1)	–	–
Total	252	(100)	690	(100)	48	(100)

Approximately 64% of events involved one case, and 8% of events involved six or more cases (Table 1). Tree fruit was the most common application target among all events (60%) and cases (46%) (Table 2). About 68% of all drift events involved a ground sprayer, 23% involved aerial application, and no other method was used more than 4% of the time (Table 3). Among tree fruit, ground sprayers—also known as orchard airblast sprayers—were involved in 89% of drift events.

**Table 3:** Spray equipment used in drift events, all crops and tree fruit only, 2000–2015

Equipment	All crops		Tree fruit	
	n	(%)	n	(%)
Ground sprayer	170	(67.5)	134	(88.7)
Aerial	58	(23.0)	13	(8.6)
Chemigation	9	(3.6)	–	–
Soil injector	4	(1.6)	–	–
Backpack sprayer	3	(1.2)	–	–
Fumigator	1	(0.4)	–	–
Dip tank or tray	1	(0.4)	1	(0.7)
Unknown	6	(2.4)	3	(2.0)
Total	252	(100)	151	(100)

Nearly 70% of cases were work-related (Table 4). At the reported time of exposure, 68% of cases were engaged in work activities not involving pesticide application. The remaining work-related cases involved handling pesticides or application equipment. Approximately 19% of cases were engaged in routine outdoor living activities. Low severity illnesses, which typically resolved without medical treatment, were recorded in 91% of cases (Supplementary Table S1) and usually included skin, eye, or upper respiratory irritation and nausea, headache, fatigue, or dizziness.

**Table 4:** Activity at time of exposure for confirmed cases, all crops and tree fruit only, 2000–2015

Activity at time of exposure	All crops		Tree fruit	
	n	(%)	n	(%)
Work-related	475	(68.8)	215	(67.2)
Work activity not involving application^a^	467	(67.7)	210	(65.6)
Handling pesticides or application equipment^b^	8	(1.2)	5	(1.6)
Not work-related (bystander)	212	(30.7)	102	(31.9)
Outdoor living activity not involving application	129	(18.7)	75	(23.4)
Indoor living activity not involving application	83	(12.0)	27	(8.4)
Unknown	3	(0.4)	3	(0.9)
Total	690	(100)	320	(100)

a. Includes exposure to field residue

b. Includes applying, mixing, or loading pesticides or repair and maintenance of application equipment

**Fig. 2:**
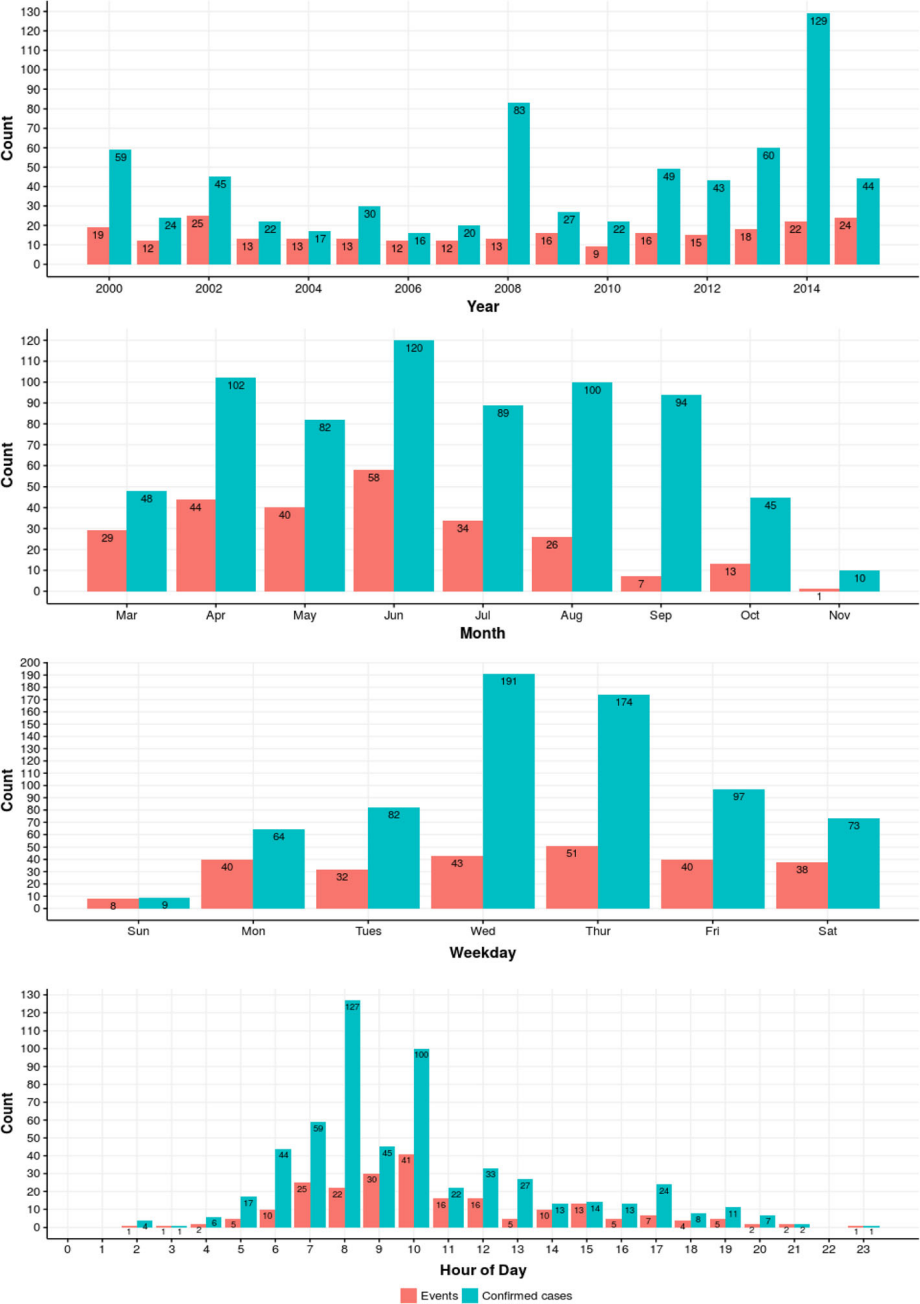
Number of drift events and confirmed cases by year, month, weekday, and hour, all crops, 2000–2015

Case counts were generally higher in more recent years, but event counts per year remained relatively constant throughout the study period. The mean number of events occurring annually was 15.8, ranging from 9 (4%) in 2010 to 25 (10%) in 2002 (Fig. 2; Supplementary Table S2). The mean number of cases occurring annually was 43.1, ranging from 16 (2%) in 2006 to 129 (19%) in 2014 (Fig. 2; Supplementary Table S2). The mean number of cases per event (event size) was 2.7 for the entire study period, with the highest years being 6.4 in 2008 and 5.9 in 2014 (Supplementary Table S2).

Figure 2 shows higher event counts (40 or more) for April through June and higher case counts (80 or more) for April through September. When restricted to tree fruit, 91% (137/151) of events and 94% (300/320) of cases occurred between March and July. Supplementary Fig. S2 shows that no tree fruit drift events occurred before the 60th day of the year (March 1 in non-leap years) or after the 290th (October 17). Tree fruit events in 2001, 2002, 2005, and 2012 happened within 120 days or fewer. There were no other clear trends related to the start or length of drift-prone periods across study years. Supplementary Fig. S3 indicates that the first week of June is a common time for drift events in tree fruit.

With the exception of Sunday, events were distributed evenly across all days of the week. The largest proportion of cases and events occurred on Wednesday (28%; 17%) and Thursday (25%; 20%). Among those with time-of-day data, 79% of cases and 74% of events occurred between 6:00 AM and 2:00 PM. Only 14 cases reported a time of exposure before 5:00 AM or after 9:00 PM. About 12% of events didn’t have reported hour of exposure data available.

The smallest distance to the application equipment (i.e. sprayer) reported by a case in a drift event had an interquartile range (IQR) of 7.3–83.8 m (24–275 ft) for all crop events (*n* = 129) and 6.4–45.7 m (21–150 ft) for tree fruit events (*n* = 79) (Table 5).

**Table 5:** Estimate of  reported distance between case receptor and sprayer source, 2000–2015

Category	Events n	Smallest distance reported by any case to sprayer (feet)				
		Minimum	25th percentile	Median	75th percentile	Maximum
All crops	129	0	23.9	75.1	274.9	5438.3
Tree fruit only	79	0	21.0	65.6	149.9	5438.3

Drift event locations were available at various levels of precision, depending primarily on event year and depth of investigation. Most events were geocoded according to street address (57%) or TRS centroid (22%) (Table 6). About 6% of events didn’t have exposure location data available and therefore could not be geocoded.

**Table 6:** Breakdown of geocoding sources

Geographic data available	n	(%)
Latitude/Longitude	15	(6.0)
Township/Range/Section centroid^a^	55	(21.8)
Street address	143	(56.7)
City centroid^a^	20	(7.9)
Zip code centroid^a^	4	(1.6)
None	15	(6.0)
Total	252	(100)

a. Centroid = geometric center of feature

**Fig. 3:**
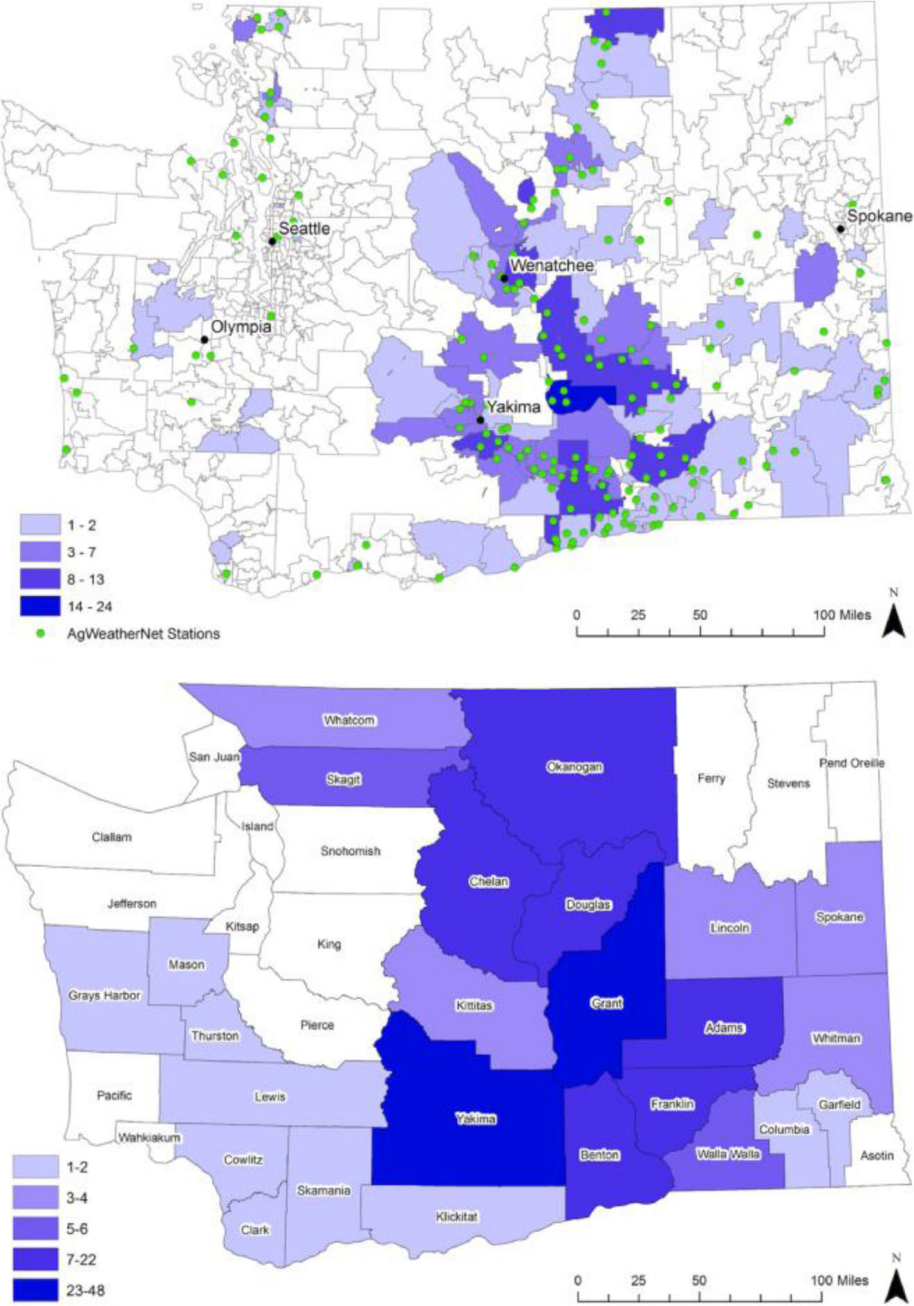
Number of events with confirmed cases by ZIP code (top panel) and county (bottom panel), all crops, 2000–2015

The proximity of AWN stations to drift events indicated good coverage in agricultural areas, especially in orchard regions (Fig. 3). Approximately 60% of events occurred in Benton, Chelan, Grant, or Yakima County, which had a combined total of 73 stations available in 2015. Approximately 92% of all crop events and 95% of tree fruit events were linked to at least one station (Table 7). Median distance from all crop events to the nearest station was 3.9 miles (6.3 km). Among events with spray records available, median distance to the nearest station was 3.6 miles (5.8 km).

**Table 7:** Distance between drift event and nearest AgWeatherNet (AWN) station, 2000–2015

Category	Events (n)	Distance to nearest station (miles)				
		Minimum	25th percentile	Median	75th percentile	Maximum
All crops	231	0.2	2.4	3.9	6.7	51.1
Tree fruit only	143	0.2	2.2	3.8	5.9	33.6
2008–2015	133	0.3	2.0	3.2	6.0	34.3
2000–2007	98	0.2	2.9	5.5	11.1	51.1
Spray records available	90	0.2	2.1	3.6	6.2	34.3
No weather station data available	21	–	–	–	–	–

In 2000, AWN was comprised of 47 stations. As a result of network expansion, the number of stations increased to 171 by 2015 (Fig. 1) despite little or no growth in the number of agricultural operations over the same time period [44]. Notably, the number of stations nearly doubled between 2007 and 2008. Among events linked to a nearby station (*n* = 231), median distance to the nearest AWN station was 5.5 miles (8.9 km) before 2008 and 3.2 miles (5.1 km) from 2008 on.

Spray records were available for 90 of 252 (36%) drift events (Table 7). Among those records, 15 had an unknown spray time, 11 had different dates for spraying and reported exposure, 5 did not report any wind conditions, and 2 did not have AWN data available at the spray start time. Records for the remaining 57 events (23% of all drift events) had wind data from both applicators and nearby AWN stations and were used in the comparative analysis.

**Fig. 4:**
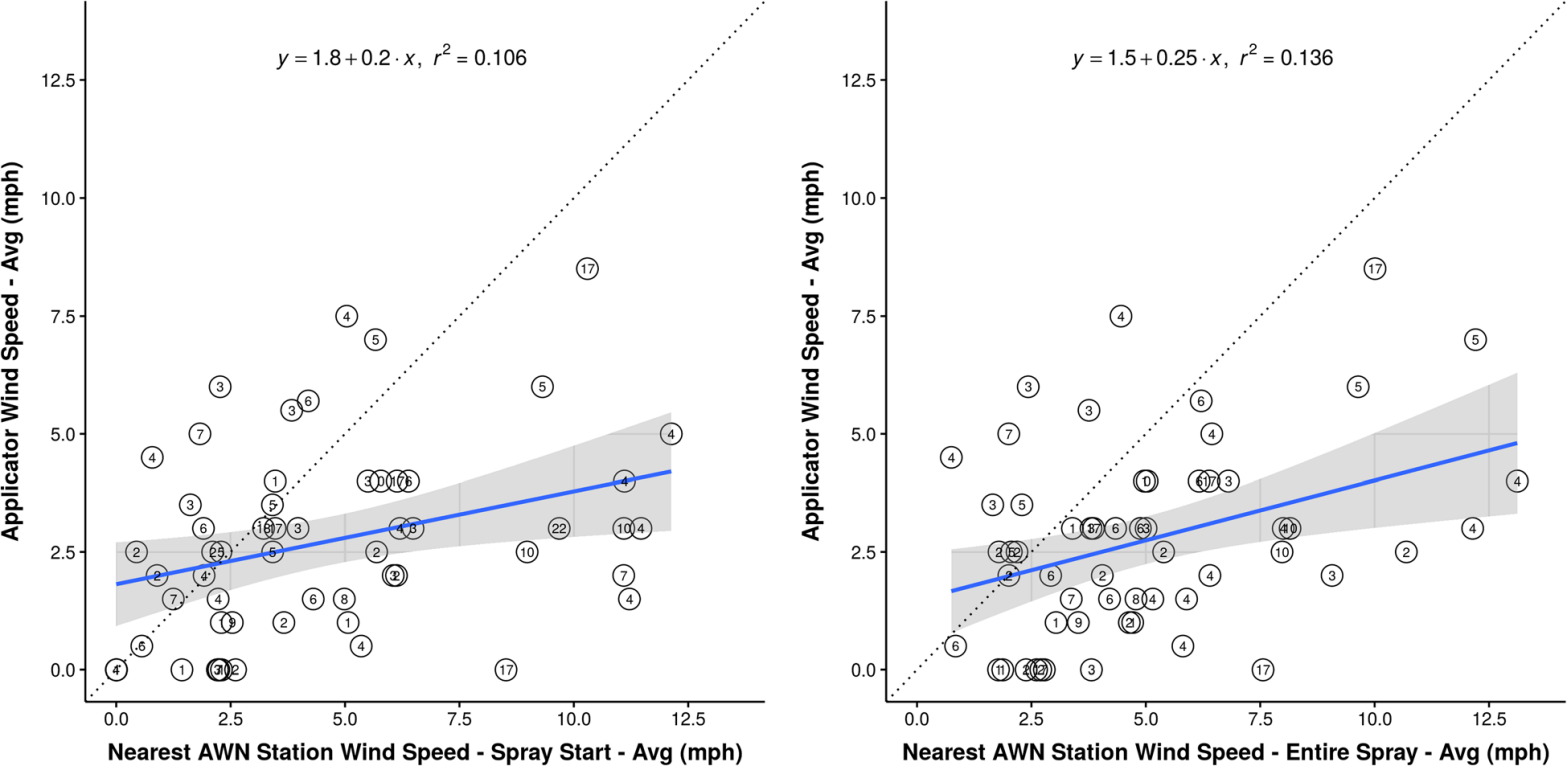
Comparison of average AgWeatherNet (AWN) and applicator-reported wind speeds in all crops (*n* = 57). Left panel (Fig. 4**a**) plots AWN wind speed average at start of spray on x-axis. Right panel (Fig. 4**b**) plots AWN wind speed average during the entire spray period on x-axis. Values in circles represent distance in miles between drift event location and nearest AWN station. The line of unity represents perfect agreement between values on the x- and y-axes. About 68% (*n* = 39) of AWN speeds at start of spray and 82% (*n* = 47) of AWN speeds during the entire spray period were higher than corresponding applicator-reported wind speeds

Applicator-reported average wind speed was weakly associated with and systematically different from average wind speed measured by the nearest AWN station. In Fig. 4a, AWN wind speed for the 15-min interval containing the spray start minute (i.e., hh:mm start time on spray record) was weakly correlated with applicator-reported wind speed (R^2^ = 0.106). About 68% (*n* = 39) of AWN wind speeds during spray start time were higher than corresponding applicator-reported wind speeds. In Fig. 4b, AWN wind speed during the entire spray period was also weakly correlated with applicator wind speed (R^2^ = 0.136). About 82% (*n* = 47) of AWN wind speeds during the entire spray period were higher than corresponding applicator wind speeds. Two-sided paired t-test results indicated that the true mean difference between applicator-reported wind speed and AWN wind speed was non-zero (Table 8). Applicator-reported wind speeds were, on average, approximately 2 mph lower than AWN wind speeds (spray start: 95% CI: − 2.84, − 1.09; *p* <  0.001; entire spray: 95%CI: − 3.01, − 1.47; *p* <  0.001).

**Table 8:** Two-sided paired t-tests for mean difference between applicator-reported wind speed and AgWeathernet (AWN) wind speed (*n*=57)

Speed comparison	Mean of differences (mph)	95% CI	*p*-value
Spray start time only			
AR-High ^a^ vs. AWN	−1.00	(− 1.99, −0.01)	0.049
AR-Avg ^b^ vs. AWN	−1.96	(−2.84, − 1.09)	< 0.001
AR-Low ^c^ vs. AWN	− 2.92	(− 3.77, − 2.07)	< 0.001
Entire spray period			
AR-High ^a^ vs. AWN	− 1.28	(− 2.16, − 0.40)	0.005
AR-Avg ^b^ vs. AWN	− 2.24	(− 3.01, − 1.47)	< 0.001
AR-Low ^c^ vs. AWN	−3.20	(− 3.96, − 2.44)	< 0.001

a. AR-High: Highest applicator-reported wind speed. On average, AR-High speed was 1.00 mph lower than 15-min average AWN speed at the time of application start (95% CI: − 1.99, − 0.01; *p* = 0.049) and 1.28 lower throughout the entire spray period (95% CI: − 2.16, − 0.40; *p* = 0.005)

b. AR-Avg: Average applicator-reported wind speed. On average, AR-Avg speed was 1.96 mph lower than 15-min average AWN speed at the time of application start (95% CI: − 2.84, − 1.09; *p* < 0.001) and 2.24 lower than AWN throughout the entire spray period (95% CI: − 3.01, − 1.47; *p* < 0.001)

c. AR-Low: Lowest applicator-reported wind speed. On average, AR-Low speed was 2.92 mph lower than 15-min average AWN speed at the time of application start (95% CI: − 3.77, − 2.07; *p* < 0.001) and 3.20 lower than AWN throughout the entire spray period (95% CI: − 3.96, − 2.44; *p* < 0.001)

## Discussion

This study examined spatial and temporal patterns of drift events and offers a better understanding of how meteorological factors contribute to pesticide drift. The linkage of epidemiological data about pesticide drift illnesses and historical weather data over multiple years is a new approach to the study of drift. Sixteen years of human pesticide incident data from 252 drift events demonstrated that drift is a recurring issue in Washington State, especially for work-related exposures from airblast applications in tree fruit. Sixty percent of all documented drift events between 2000 and 2015 occurred in four counties, where weather data were collected from 73 different weather stations. A comparison of applicator-reported and AWN wind data yielded insights about field-based practices, our “nearest neighbor” approach to using weather network data, and recommendations for understanding and reducing drift. This work could lead to new training materials that improve the practice of pesticide application and better documentation of spray drift events. Both Washington State and USEPA would like to offer farm managers and workers better guidance in the area of pesticide drift prevention [45, 46].

Managers of farms and orchards are required to maintain records of pesticide applications. However, missing or incomplete spray records limited our comparison of self-reported wind speed to AWN values. Sixty-four percent of drift events had no spray records available. Records for an additional 13% of spray events had incomplete information. Thus, a total of 77% of drift events between 2000 and 2015 didn’t have sufficient spray record information to fully evaluate spray drift. For these events, adequate information was available from state investigation reports from the Washington Departments of Agriculture (WSDA) and Labor & Industries (L&I) to meet the WADOH epidemiological case definition, which does not require meteorological conditions.

The lack of information available from spray records is of concern to investigators who examine cases of illness resulting from drift. Spray records are a component of WSDA regulatory investigations and enhance data compiled by other state agencies such as worker health and safety at L&I and WADOH. Spray records are essential to the ability of WADOH to “secure any and all such information as may be necessary to adequately determine the nature and causes of any case of pesticide poisoning” [5]. For the 3 year period of 2000–2002, 84% of the spray records for WADOH drift investigations were incomplete or unavailable to the investigator. That percentage decreased to 55% for the 3 year period of 2013–2015. This demonstrates improvement in the ability of WADOH to obtain complete spray records. When spray records are incomplete or unavailable, WADOH investigators rely on WSDA confirmation of pesticide type, date, and location of application.

Our results provide actionable information about drift events in terms of time, space, and wind variability. Weather station network data are viewable through an online portal, which could enable alerts for large changes in wind speed or direction in the last 15 min. To maximize the potential for actionable information, applicators could theoretically receive alerts at higher time resolutions (e.g. 5 or 30 s) by linking to the nearest weather station using new technologies such as long-range (LoRa) networking. A key consideration of this study is motivating the need for installing “hyperlocal” meteorological sensors with continuous readouts rather than using a handheld anemometer once at the beginning of a spray.

One hypothesis about the 2014 spike in drift events was that several occurred early in the growing season when orchard tree canopies were still dormant or immature, raising the possibility that overspray occurred due to using sprayers calibrated for full canopy trees. Messages about application best practices and exposure prevention can be delivered to managers, crew supervisors, and workers shortly before annual increases in pesticide use that begin in March. The drift-prone period of March–July, which increases demands on state investigators, is an appropriate time to boost capacity for public health surveillance. Resources could focus on those counties with the highest number of drift events.

The smallest distance reported by drift cases to the sprayer source provided important evidence about the US EPA’s Application Exclusion Zone (AEZ). The AEZ regulation states that orchard airblast sprayers must be free of all untrained persons within a 100-ft (30.5 m) radius during pesticide applications [47]. If the AEZ were in place during the study period (including off-site locations), we estimate up to half of all drift events involving pesticide exposure might have been prevented. Our recent studies of orchard airblast sprayers have demonstrated that spray drift can extend beyond the boundary defined by the AEZ [14–17], which is an expected outcome for standard regulatory drift modeling. Our studies also demonstrated that wind speed and direction can be well characterized with on-site meteorological stations.

Findings from this study are similar to others, especially with regard to incidence rates, maximum wind speeds, and distance from the sprayer. Between 2010 and 2015, the incidence rate of agricultural drift illnesses in Washington ranged from 0.33 to 1.85 cases per 100,000 individuals [48]. Lee et al. [9] found a slightly lower incidence rate for 11 states between 1998 and 2006. Incidence rates ranged from 0.139 to 0.532 cases per 100,000 individuals. In an event involving 20 orchard workers in 2014, applicator and meteorological records showed that wind speeds were low (0–4 mph, 0–1.8 m/s) early on a spray day, but that wind speeds increased to 18 mph (8.0 m/s) during the time of exposure later in the day [20]. This finding is consistent with five events from our study that had AWN wind speed readings above 20 mph (8.9 m/s), one of which reached 24 mph (10.8 m/s). Lee et al. [9] also reported that occupational cases represented 68% of cases exposed within 0.25 miles (0.4 km) of the application site and non-occupational cases represented 73% of cases exposed more than 0.25 miles (0.4 km) away. These findings matched our own in terms of occupationally-related events, only one of which was more than 0.25 miles (0.4 km). However, our data about minimum reported distance didn’t show illnesses among non-occupational events further than 0.25 miles (0.4 km).

There were several limitations to this study that mainly relate to space and time. Regarding space, locations were not always precise and average distances between weather stations and drift events were large at times. Although we used geocoordinate points in our analysis, administrative areas such as cities, zip codes, and TRS code centroids were sometimes imprecise. When spray records were available, we occasionally had to rely on the “Address of Person for Whom Pesticide was Applied” field, which could have been the location of a separate administrative building away from the application site—although we used satellite imagery to confirm locations. Additionally, using data from a station located nearly 4 miles (6.3 km) away was unlikely to capture wind conditions experienced across diverse terrain and microclimates. That stated, distance to nearby station didn’t appear to have a strong impact on our findings. Distance to the nearest AgWeatherNet (AWN) station had a weak positive association with AWN station wind speed (R^2^ = 0.105) and no clear association with applicator-reported wind speed (R^2^ = 0.0225) (Supplementary Fig. S4). In anticipation of geographic uncertainties, we analyzed mean wind speed when using the nearest station only (4.7 mph) versus the nearest ten stations (4.9 mph) and found that the difference was only 0.2 mph (0.09 m/s) (Supplementary Table S3). The range of reported distances was due to discrepancies between reports (e.g., applicators vs. exposed individuals) or multiple individuals whose distances varied from one sprayer source. Distance was most often gathered from the narratives in the WADOH and WSDA reports. Sometimes, distance was reported as the number of tree rows between the applicator and exposed individuals; in this case, we used a row width of 8 ft. Concerning limitations of time, AWN stations and applicator wind speeds used different timescales and therefore may not be directly comparable. Using 15-min average wind readings in the absence of wind gust data, which weren’t available for this analysis, could have muted the true effect of wind speed changes. Furthermore, the influence of other meteorological variables, such as humidity and temperature, weren’t evaluated in this study. Investigation narratives between 2012 and 2015 indicated a possible role for temperature inversion in at least five drift events, but more systematic evaluation is needed. This study focused on wind velocity, which typically changes more frequently than temperature and humidity over the course of several hours.

There are several avenues to explore for future research. For our study, we used a “nearest neighbor” geoprocessing approach to estimate wind conditions for each drift event [49, 50]. More sophisticated approaches could include data from several nearby stations and analyze differences in topographical variation. Venäläinen [51] and Luo et al. [52] have described successful kriging methods for using a network of weather stations to interpolate measured weather data onto a grid despite the challenges of spatializing meteorological data in heterogeneous landscapes. Researchers can use gridded data to run agricultural models, such as crop yield or pest life cycles [51]. Future studies could use cokriging (wind speed) and anisotropic kriging (wind direction) to generate estimates across a grid appropriate for AgWeatherNet wind data [52].

Internal and external validation of a weather station network represents another avenue for future research. Studies of this kind could provide information about when it’s reasonable to use data from the nearest station. Further analyses should implement geographical features such as elevation profiles between drift event locations and weather stations. We would expect regions with more elevation and microclimate variability to be less reliable predictors of drift events than in comparatively flat areas.

We also view the concept of “wind ramping” as a useful tool for prediction of future drift events. Wind ramping is defined as large shifts in wind speed at a given location over a short period of time [53–58]. As we will discuss in a subsequent paper, analysis of wind ramping could assist with estimating the risk of drift during periods of spraying and non-spraying. Table 9 provides a summary of wind ramping characteristics during drift events with applicator records.

**Table 9:** Wind ramping characteristics during drift events with applicator records, AgWeatherNet (AWN) data, 2000–2015

Spray window	Length (hours)	Drift events (n)	Wind speed (mph)				Wind direction (degrees)			Both ramps^b^
			AM	SD	CV	Ramp events^a^	SD	Range	Ramp events^a^	
Short	0.25–1.75	20	4.43	1.50	33.8	8	0.68	77.6	3	2
Medium	2.00–7.00	19	5.74	1.53	26.6	11	0.81	129.1	4	3
Long	7.25–15.75	18	4.78	1.79	37.5	13	1.16	232.0	9	7
Total	0.25–15.75	57	4.98	1.61	32.3	32	0.90	143.5	16	12

*AM* Arithmetic mean; *SD* Standard deviation; *CV* Coefficient of variation

a. A ramp event was defined as a drift event spray window with a wind speed or wind direction standard deviation greater than 1

b. Indicates the number of drift events that had both a wind speed ramp and a wind direction ramp

Maintenance of accurate spray records is essential for characterizing drift events. State agencies can work closely with pesticide users to improve the quality of such records, providing training to both licensed and unlicensed applicators. We recommend that applicators continue to be given clear instructions and standardized methods to record such information.

Pesticide applicators would also benefit from better information about meteorological conditions at the application site. Tradeoffs need to be analyzed between the reliability of using an on-site hand-held anemometer once at the beginning of a spray versus a “neighboring” weather station with continuous readout. Wind speed at a given location usually decreases when measured closer to the ground, so future recommendations should include the most appropriate height(s) for taking measurements relevant for spray plumes that reach treetops. Relatively low-cost weather stations could be placed on-site or on-tractor, and information could be transmitted in real-time through connection with a mobile device or digital readout on the tractor. This approach would lead naturally to electronic record keeping. Alternatively, applicators could access the nearest AWN station before and during applications to ensure that wind speeds and directions are appropriate for spraying. Our understanding is that AWN continues to grow and expand its network. Eventually, this will reduce the average distance to the nearest station for each spray operation. The most geographically relevant site(s) should be chosen for spray decisions related to meteorology, which typically means “hyperlocal” readings that may or may not be part of a network. We recommend that the state sponsor a pilot program to evaluate the most practical means of providing applicators with training about real-time meteorological information.

Washington agriculture is diverse, productive, and a large component of the State’s economy [59, 60]. Many agricultural producers have embraced “precision agriculture” as a means of improving efficiency through development of accurate and time-sensitive information. The National Academy of Sciences (NAS) defines precision agriculture as “a management strategy that uses information technologies to bring data from multiple sources to bear on decisions associated with crop production” [61]. The NAS further states that precision agriculture offers “the promise of increasing productivity while decreasing production costs and minimizing environmental impacts.” An excellent example of a precision agriculture tool in Washington State is the Washington State University Decision Aid System [30]: “WSU-DAS [das.wsu.edu] is a web-based platform designed to transfer time-sensitive information to decision-makers in the tree fruit industry.” We applaud this advance in agricultural production and recommend that the goals of precision agriculture be extended to issues of health and safety. There is no technological barrier to providing pesticide applicators with “time-sensitive information” to make decisions regarding appropriate meteorological conditions for spraying. We hope that agricultural producers, as well as federal and state agencies, will work to upgrade the quality and timeliness of information that can be used to prevent or minimize pesticide drift.

## Conclusions

Drift events result from a complex array of factors in the agricultural setting. Our study attempted to shed light on such events by utilizing the known spatio-temporal aspects of drift and historical weather data. Particularly critical for this analysis is more accurate and complete information about location, time, wind speed, and wind direction. Our findings can be incorporated into new training materials to improve the practice of pesticide application and for better documentation of spray drift events. A precision agriculture approach offers technological solutions that simplify the task of tracking pesticide use and weather conditions. Public health investigators will benefit greatly from improved meteorological data and accurate application records, as well as from the explanatory and predictive potential of wind ramping studies for growers and surrounding communities.

## Supplementary Information

**Additional file 1.**

## Data Availability

The human illness data that support the findings of this study are available from the Washington Tracking Network but restrictions apply to the availability of these data, which were used under data use agreement to protect personal identifiers. Weather data were provided courtesy of and copyright by Washington State University AgWeatherNet, which are publicly available through an online portal.
