# Supplementary material for: Examining the role of wind in human illness due to pesticide drift in Washington state, 2000–2015
Source: Environ Health. 2021 Mar 15;20:26. doi: 10.1186/s12940-021-00693-3 (PMC7958705; doi:10.1186/s12940-021-00693-3)

**SUPPLEMENTARY MATERIAL**


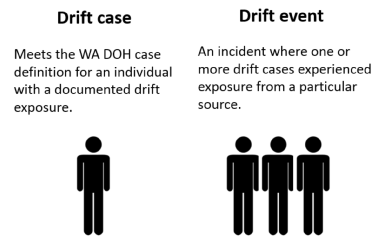


**Supplementary Figure S1.** Definition of a drift case and drift event.


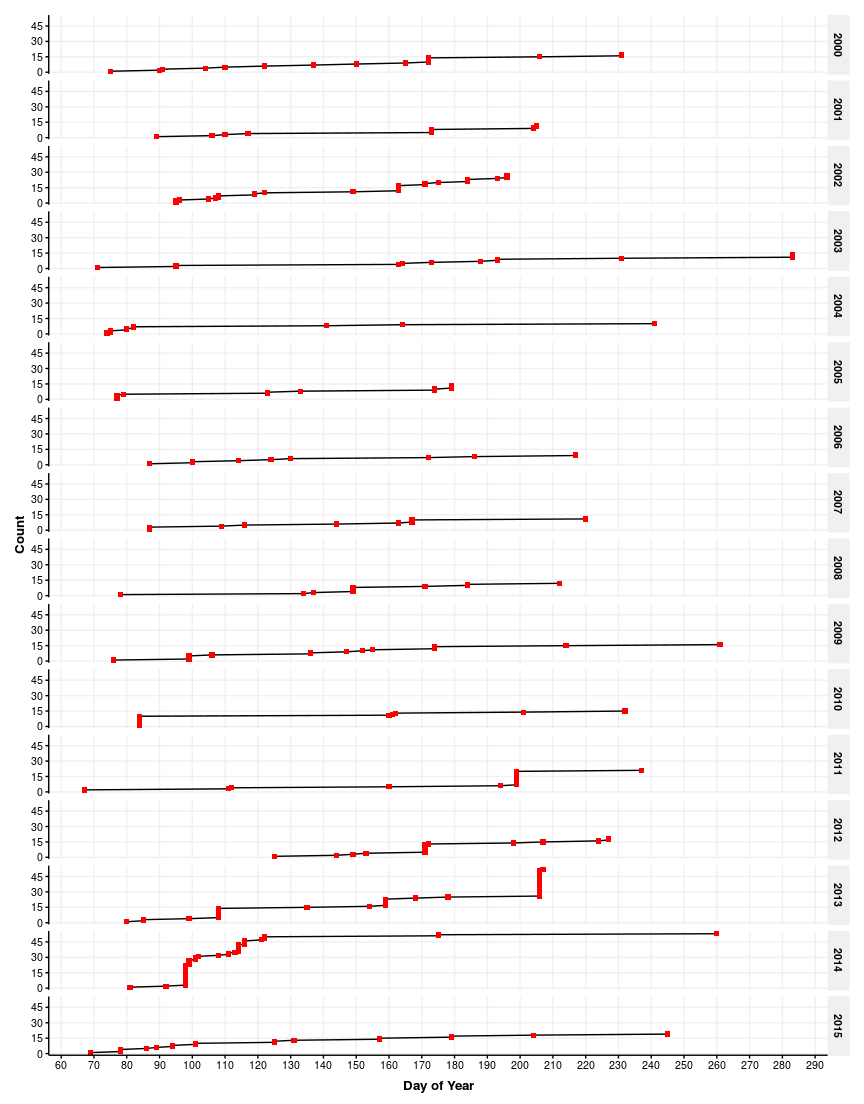


**Supplementary Figure S2.** Drift cases (n=320) by day of the year, tree fruit only, 2000-2015. Each confirmed drift case is plotted as a red mark on the day of occurrence in an annual cumulative total.


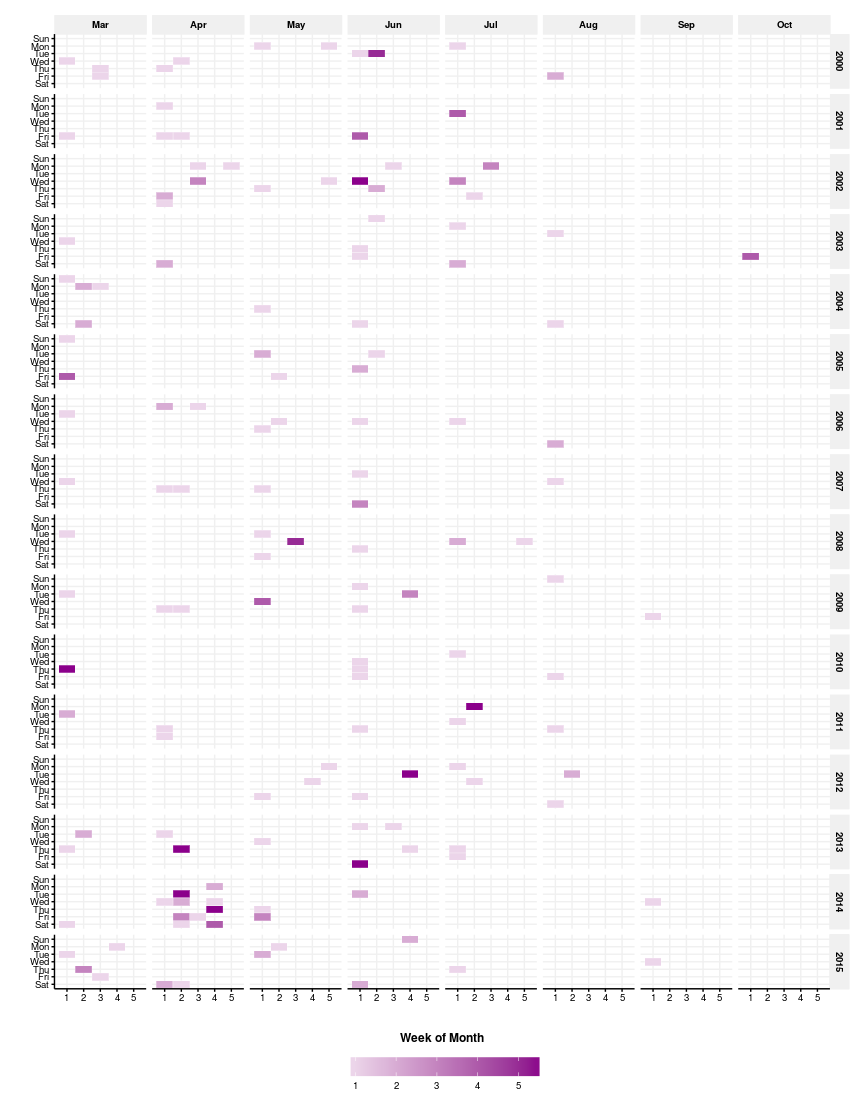


**Supplementary Figure S3.** Drift events (n=151) by calendar day, tree fruit only, 2000-2015. Color indicates number of cases per event. Events with 5+ cases are deepest purple.

**
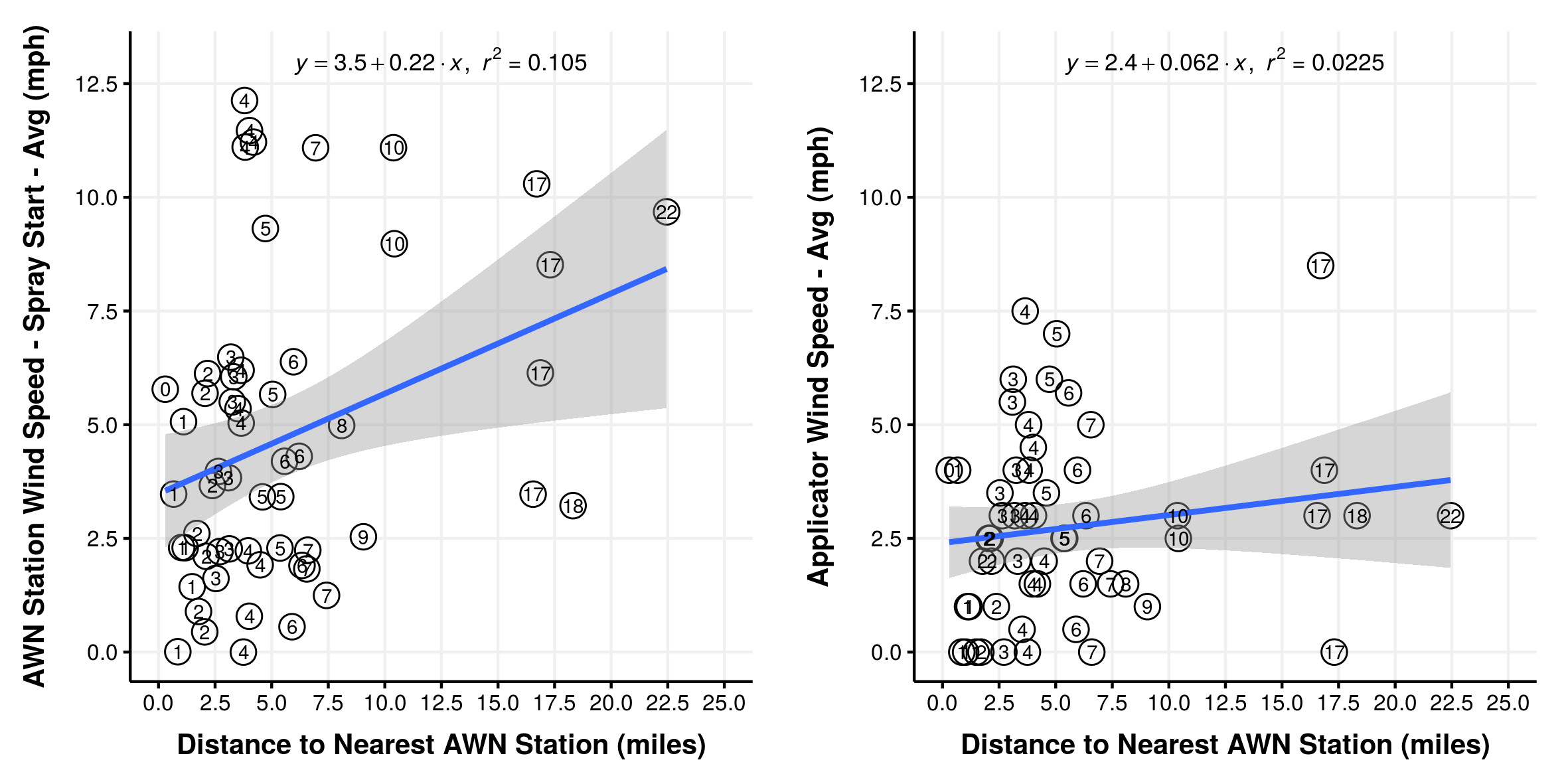
Supplementary Figure S4.** Among 57 all crop drift events at the time of spray start, distance to the nearest AgWeatherNet (AWN) station had a weak positive association with AWN station wind speed, but not with applicator self-reported wind speed (mph).

| **Supplementary Table S1.** Severity of illness for confirmed cases, all crops and tree fruit only, 2000-2015. Illness severity was categorized into low, moderate, and high using criteria developed by the SENSOR-Pesticides program. | | |
| --- | --- | --- |
| Severity of illness or injury | All crops  n (%) | Tree fruit  n (%) |
| High^a^ | 2 (0.3) | 1 (0.3) |
| Moderate^b^ | 58 (8.4) | 3 (10.3) |
| Low^c^ | 628 (91.0) | 84 (88.8) |
| Unlikely, insufficient, asymptomatic, unrelated | 1 (0.1) | 1 (0.3) |
| Unknown | 1 (0.1) | 1 (0.3) |
| Total | 690 (100) | 20 (100) |
| a. High severity: life threatening and could have resulted in permanent disability.  b. Moderate severity: involved systemic manifestations and required treatment.  c. Low severity: typically resolved without treatment and included skin, eye or upper respiratory irritation and fever, headache, fatigue, or dizziness. | | |

| **Supplementary Table S2.** Year of drift event by case status, all crops, 2000-2015. | | | | |
| --- | --- | --- | --- | --- |
| Year | Events  n (%) | Confirmed cases  n (%) | Unconfirmed cases n (%) | Mean event size^a^ |
| 2000 | 19 (7.5) | 59 (8.6) | 1 (2.1) | 3.1 |
| 2001 | 12 (4.8) | 24 (3.5) | - | 2.0 |
| 2002 | 25 (9.9) | 45 (6.5) | 2 (4.2) | 1.8 |
| 2003 | 13 (5.2) | 22 (3.2) | - | 1.7 |
| 2004 | 13 (5.2) | 17 (2.5) | - | 1.3 |
| 2005 | 13 (5.2) | 30 (4.3) | - | 2.3 |
| 2006 | 12 (4.8) | 16 (2.3) | - | 1.3 |
| 2007 | 12 (4.8) | 20 (2.9) | - | 1.7 |
| 2008 | 13 (5.2) | 83 (12.0) | - | 6.4 |
| 2009 | 16 (6.3) | 27 (3.9) | - | 1.7 |
| 2010 | 9 (3.6) | 22 (3.2) | - | 2.4 |
| 2011 | 16 (6.3) | 49 (7.1) | 14 (29.2) | 3.1 |
| 2012 | 15 (6.0) | 43 (6.2) | 2 (4.2) | 2.9 |
| 2013 | 18 (7.1) | 60 (8.7) | 9 (18.8) | 3.3 |
| 2014 | 22 (8.7) | 129 (18.7) | 6 (12.5) | 5.9 |
| 2015 | 24 (9.5) | 44 (6.4) | 14 (29.2) | 1.8 |
| Total | 252 (100) | 690 (100) | 48 (100) | 2.7 |
| Mean | 15.8 | 43.1 | 3 |  |
| Median | 14.0 | 36.5 | 0 |  |
| a. Mean event size was computed by dividing the number of confirmed cases by the number of  events in a given year | | | | |

| **Supplementary Table S3.** Wind conditions at spray start time for each drift event according to nearby weather stations and applicator report, 2000-2015. | | | | | | | | | | | | | | | | | | | | | | | |
| --- | --- | --- | --- | --- | --- | --- | --- | --- | --- | --- | --- | --- | --- | --- | --- | --- | --- | --- | --- | --- | --- | --- | --- |
| **Drift Event** | |  | **All Nearby Stations** | | | | | | | | |  | **Nearest Station Only** | | | | |  | **Applicator Spray Record** | | | | |
|  | Year |  |  | Distance |  | Wind speed | | |  | Wind direction | |  | Distance |  | Wind speed |  | Wind direction |  | Wind speed | | |  | Wind direction |
| # |  |  | # Stations | AM (mi) |  | AM  (mph) | SD  (mph) | CV  (%) |  | Range  (degrees) | SD  (degrees) |  | (mi) |  | (mph) |  | (from) |  | Low (mph) | Average (mph) | High (mph) |  | (from) |
| 1 | 2000 |  | 3 | 6.9 |  | 1.2 | 0.2 | 19.4 |  | 197.2 | 1.7 |  | 1.5 |  | 1.4 |  | SSW |  | 0 | 0 | 0 |  | - |
| 2 | 2001 |  | 2 | 12.0 |  | 3.4 | 0.1 | 4.1 |  | 56.0 | 0.5 |  | 0.7 |  | 3.5 |  | NNW |  | 2 | 4 | 6 |  | SE |
| 3 | 2001 |  | 1 | 17.3 |  | 8.5 | - | - |  | 0.0 | 0.0 |  | 17.3 |  | 8.5 |  | SSW |  | 0 | 0 | 0 |  | - |
| 4 | 2002 |  | 1 | 4.0 |  | 0.8 | - | - |  | 0.0 | 0.0 |  | 4.0 |  | 0.8 |  | SW |  | 1 | 4.5 | 8 |  | S |
| 5 | 2006 |  | 1 | 18.3 |  | 3.2 | - | - |  | 0.0 | 0.0 |  | 18.3 |  | 3.2 |  | W |  | 3 | 3 | 3 |  | NW |
| 6 | 2006 |  | 2 | 8.8 |  | 3.4 | 0.0 | 0.8 |  | 5.4 | 0.0 |  | 5.4 |  | 3.4 |  | N |  | 0 | 2.5 | 5 |  | NW |
| 7 | 2007 |  | 8 | 16.6 |  | 1.0 | 0.6 | 64.5 |  | 220.1 | 1.3 |  | 2.0 |  | 0.4 |  | NE |  | 0 | 2.5 | 5 |  | S |
| 8 | 2007 |  | 2 | 4.5 |  | 1.3 | 1.1 | 81.5 |  | 80.3 | 0.7 |  | 2.1 |  | 2.1 |  | ENE |  | 2.5 | 2.5 | 2.5 |  | S |
| 9 | 2007 |  | 2 | 22.7 |  | 5.8 | 5.5 | 93.6 |  | 16.4 | 0.1 |  | 22.4 |  | 9.7 |  | WNW |  | 2 | 3 | 4 |  | SW |
| 10 | 2007 |  | 3 | 6.1 |  | 8.0 | 5.1 | 63.7 |  | 104.5 | 0.9 |  | 4.2 |  | 11.2 |  | WNW |  | 0 | 1.5 | 3 |  | E |
| 11 | 2007 |  | 2 | 7.5 |  | 7.5 | 5.0 | 66.6 |  | 12.5 | 0.1 |  | 6.9 |  | 11.1 |  | WNW |  | 1 | 2 | 3 |  | W |
| 12 | 2008 |  | 6 | 10.7 |  | 4.1 | 2.7 | 66.4 |  | 157.7 | 1.0 |  | 3.7 |  | 0.0 |  | S |  | 0 | 0 | 0 |  | W |
| 13 | 2008 |  | 3 | 17.3 |  | 4.2 | 4.2 | 98.4 |  | 11.7 | 0.1 |  | 10.4 |  | 9.0 |  | WSW |  | 0 | 2.5 | 5 |  | N |
| 14 | 2009 |  | 9 | 7.5 |  | 3.6 | 2.1 | 57.8 |  | 169.8 | 1.2 |  | 2.4 |  | 3.7 |  | ESE |  | 0 | 1 | 2 |  | NE |
| 15 | 2009 |  | 9 | 10.1 |  | 3.1 | 1.9 | 60.9 |  | 199.0 | 1.0 |  | 2.2 |  | 6.1 |  | WSW |  | 2 | 2 | 2 |  | W |
| 16 | 2009 |  | 9 | 12.2 |  | 5.3 | 1.1 | 20.5 |  | 87.6 | 0.4 |  | 3.7 |  | 5.0 |  | E |  | 5 | 7.5 | 10 |  | SE |
| 17 | 2009 |  | 9 | 18.4 |  | 2.1 | 1.1 | 53.5 |  | 187.9 | 1.4 |  | 4.0 |  | 2.2 |  | W |  | 0 | 1.5 | 3 |  | W |
| 18 | 2010 |  | 10 | 10.3 |  | 6.6 | 3.0 | 46.2 |  | 104.3 | 0.6 |  | 3.3 |  | 6.1 |  | WSW |  | 2 | 2 | 2 |  | NW |
| 19 | 2010 |  | 9 | 21.8 |  | 2.7 | 3.6 | 132.0 |  | 242.8 | 1.3 |  | 6.6 |  | 1.8 |  | NNE |  | 5 | 5 | 5 |  | W |
| 20 | 2011 |  | 10 | 10.1 |  | 13.0 | 1.8 | 13.6 |  | 48.2 | 0.3 |  | 4.7 |  | 9.3 |  | W |  | 6 | 6 | 6 |  | SW |
| 21 | 2011 |  | 8 | 15.3 |  | 8.3 | 2.6 | 31.5 |  | 234.3 | 1.2 |  | 3.6 |  | 6.2 |  | WNW |  | 0 | 3 | 6 |  | W |
| 22 | 2011 |  | 10 | 14.9 |  | 3.8 | 2.0 | 52.7 |  | 199.6 | 1.2 |  | 2.5 |  | 1.6 |  | WNW |  | 3 | 3.5 | 4 |  | SW |
| 23 | 2011 |  | 5 | 32.7 |  | 7.6 | 1.7 | 22.0 |  | 205.3 | 1.1 |  | 8.1 |  | 5.0 |  | SSE |  | 0 | 1.5 | 3 |  | SSE |
| 24 | 2011 |  | 9 | 15.5 |  | 2.9 | 1.7 | 56.3 |  | 252.2 | 1.8 |  | 7.4 |  | 1.2 |  | ESE |  | 0 | 1.5 | 3 |  | W |
| 25 | 2011 |  | 10 | 13.8 |  | 2.9 | 2.1 | 72.9 |  | 235.4 | 1.2 |  | 2.7 |  | 2.2 |  | SSW |  | 0 | 0 | 0 |  | - |
| 26 | 2011 |  | 8 | 10.1 |  | 1.6 | 2.2 | 137.0 |  | 207.9 | 0.9 |  | 1.8 |  | 0.9 |  | SW |  | 1 | 2 | 3 |  | W |
| 27 | 2012 |  | 6 | 33.7 |  | 4.3 | 1.2 | 27.6 |  | 105.2 | 0.9 |  | 16.6 |  | 3.5 |  | SW |  | 3 | 3 | 3 |  | NNE |
| 28 | 2012 |  | 9 | 13.4 |  | 10.4 | 3.8 | 36.7 |  | 79.5 | 0.5 |  | 3.5 |  | 5.4 |  | W |  | 0.5 | 0.5 | 0.5 |  | NW |
| 29 | 2012 |  | 10 | 14.4 |  | 2.5 | 1.5 | 59.1 |  | 255.4 | 1.8 |  | 0.9 |  | 0.0 |  | NNE |  | 0 | 0 | 0 |  | - |
| 30 | 2012 |  | 10 | 10.3 |  | 13.2 | 3.7 | 27.9 |  | 43.3 | 0.2 |  | 3.8 |  | 11.1 |  | SSW |  | 4 | 4 | 4 |  | SE |
| 31 | 2012 |  | 10 | 25.0 |  | 2.6 | 1.7 | 66.5 |  | 281.5 | 1.6 |  | 1.0 |  | 2.3 |  | ESE |  | 0 | 0 | 0 |  | - |
| 32 | 2012 |  | 6 | 38.2 |  | 5.2 | 1.8 | 34.6 |  | 107.7 | 0.6 |  | 16.9 |  | 6.1 |  | N |  | 3 | 4 | 5 |  | N |
| 33 | 2012 |  | 7 | 68.9 |  | 2.2 | 2.5 | 117.0 |  | 283.0 | 1.9 |  | 5.9 |  | 0.6 |  | E |  | 0 | 0.5 | 1 |  | E |
| 34 | 2012 |  | 10 | 9.7 |  | 2.6 | 2.4 | 92.2 |  | 252.8 | 1.3 |  | 5.4 |  | 2.3 |  | SSW |  | 1 | 2.5 | 4 |  | NW |
| 35 | 2012 |  | 8 | 15.0 |  | 3.8 | 1.5 | 40.0 |  | 185.0 | 1.1 |  | 3.1 |  | 2.3 |  | ENE |  | 5 | 6 | 7 |  | W |
| 36 | 2013 |  | 9 | 13.9 |  | 7.2 | 3.9 | 54.1 |  | 174.7 | 0.9 |  | 3.1 |  | 3.8 |  | NNW |  | 3 | 5.5 | 8 |  | NW |
| 37 | 2013 |  | 10 | 20.7 |  | 4.0 | 2.0 | 50.1 |  | 267.1 | 1.5 |  | 6.0 |  | 6.4 |  | S |  | 4 | 4 | 4 |  | N |
| 38 | 2013 |  | 9 | 9.8 |  | 2.6 | 0.9 | 33.0 |  | 261.9 | 1.4 |  | 1.2 |  | 2.3 |  | NE |  | 1 | 1 | 1 |  | S |
| 39 | 2013 |  | 9 | 13.1 |  | 4.9 | 1.6 | 32.4 |  | 243.5 | 1.2 |  | 6.2 |  | 4.3 |  | WSW |  | 1 | 1.5 | 2 |  | SW |
| 40 | 2013 |  | 9 | 9.8 |  | 5.3 | 0.9 | 16.1 |  | 188.8 | 1.1 |  | 1.1 |  | 5.1 |  | SE |  | 1 | 1 | 1 |  | N |
| 41 | 2013 |  | 10 | 12.0 |  | 5.5 | 2.4 | 42.8 |  | 236.1 | 1.3 |  | 4.6 |  | 3.4 |  | SE |  | 3 | 3.5 | 4 |  | - |
| 42 | 2013 |  | 10 | 13.2 |  | 3.4 | 2.6 | 74.5 |  | 225.2 | 1.6 |  | 1.7 |  | 2.6 |  | SW |  | 0 | 0 | 0 |  | - |
| 43 | 2013 |  | 9 | 26.6 |  | 2.4 | 2.2 | 91.0 |  | 253.9 | 1.9 |  | 6.6 |  | 2.2 |  | N |  | 0 | 0 | 0 |  | - |
| 44 | 2014 |  | 9 | 17.5 |  | 3.5 | 1.5 | 41.6 |  | 262.8 | 1.5 |  | 4.5 |  | 1.9 |  | ENE |  | 0 | 2 | 4 |  | SW |
| 45 | 2014 |  | 9 | 17.7 |  | 5.0 | 3.5 | 69.9 |  | 262.6 | 2.3 |  | 3.8 |  | 12.1 |  | NNW |  | 5 | 5 | 5 |  | NE |
| 46 | 2014 |  | 8 | 15.2 |  | 4.3 | 1.8 | 41.2 |  | 245.7 | 1.3 |  | 5.0 |  | 5.7 |  | SW |  | 3 | 7 | 11 |  | WNW |
| 47 | 2014 |  | 9 | 15.1 |  | 6.6 | 4.0 | 60.9 |  | 152.4 | 0.7 |  | 10.4 |  | 11.1 |  | NW |  | 3 | 3 | 3 |  | SW |
| 48 | 2014 |  | 10 | 14.5 |  | 5.2 | 2.0 | 39.0 |  | 193.6 | 0.8 |  | 2.1 |  | 5.7 |  | NW |  | 0 | 2.5 | 5 |  | NW |
| 49 | 2014 |  | 10 | 9.5 |  | 2.9 | 2.5 | 85.3 |  | 279.4 | 1.6 |  | 6.3 |  | 1.9 |  | SSE |  | 2 | 3 | 4 |  | E |
| 50 | 2014 |  | 9 | 12.3 |  | 9.6 | 3.1 | 32.7 |  | 106.9 | 0.5 |  | 4.0 |  | 11.5 |  | S |  | 2 | 3 | 4 |  | SE |
| 51 | 2014 |  | 10 | 14.7 |  | 5.2 | 2.6 | 50.1 |  | 219.3 | 1.2 |  | 2.6 |  | 4.0 |  | SSE |  | 3 | 3 | 3 |  | - |
| 52 | 2014 |  | 9 | 35.8 |  | 10.9 | 3.6 | 32.7 |  | 51.5 | 0.3 |  | 16.7 |  | 10.3 |  | WSW |  | 7 | 8.5 | 10 |  | SW |
| 53 | 2014 |  | 9 | 27.0 |  | 6.5 | 2.4 | 36.7 |  | 94.7 | 0.6 |  | 0.3 |  | 5.8 |  | WSW |  | 0 | 4 | 8 |  | W |
| 54 | 2014 |  | 10 | 16.0 |  | 4.7 | 4.8 | 103.5 |  | 242.0 | 1.3 |  | 5.6 |  | 4.2 |  | NW |  | 4.4 | 5.7 | 7 |  | - |
| 55 | 2015 |  | 10 | 10.8 |  | 5.1 | 0.7 | 13.8 |  | 79.4 | 0.4 |  | 3.3 |  | 5.5 |  | SE |  | 3 | 4 | 5 |  | NW |
| 56 | 2015 |  | 9 | 15.5 |  | 4.4 | 1.7 | 39.4 |  | 233.2 | 2.0 |  | 9.1 |  | 2.5 |  | W |  | 1 | 1 | 1 |  | WSW |
| 57 | 2015 |  | 10 | 7.0 |  | 6.5 | 2.4 | 36.6 |  | 112.7 | 0.6 |  | 3.2 |  | 6.5 |  | WSW |  | 3 | 3 | 3 |  | WSW |
|  | Minimum |  | 1.0 | 4.0 |  | 0.8 | 0.0 | 0.8 |  | 0.0 | 0.0 |  | 0.3 |  | 0.0 |  |  |  | 0.0 | 0.0 | 0.0 |  |  |
|  | 25^th^ %ile |  | 6.0 | 10.1 |  | 2.9 | 1.5 | 32.8 |  | 87.6 | 0.5 |  | 2.5 |  | 2.2 |  |  |  | 0.0 | 1.5 | 2.0 |  |  |
|  | Mean |  | 7.6 | 16.2 |  | 4.9 | 2.3 | 53.6 |  | 161.7 | 1.0 |  | 5.5 |  | 4.7 |  |  |  | 1.8 | 2.7 | 3.7 |  |  |
|  | Median |  | 9.0 | 14.4 |  | 4.3 | 2.1 | 50.1 |  | 188.8 | 1.1 |  | 4.0 |  | 3.8 |  |  |  | 1.0 | 2.5 | 3.0 |  |  |
|  | 75^th^ %ile |  | 10.0 | 17.5 |  | 6.5 | 3.0 | 66.5 |  | 242.0 | 1.3 |  | 6.3 |  | 6.1 |  |  |  | 3.0 | 4.0 | 5.0 |  |  |
|  | Maximum |  | 10.0 | 68.9 |  | 13.2 | 5.5 | 137.0 |  | 283.0 | 2.3 |  | 22.4 |  | 12.1 |  |  |  | 7.0 | 8.5 | 11.0 |  |  |
|  | a. AM: arithmetic mean, SD: standard deviation, CV: coefficient of variation  b. Station data is 15-minute average measurement that included the minute when application started as reported on spray records | | | | | | | | | | | | | | | | | | | | | | |

**Time Series Plots for Events in Supplementary Table S4.** Comparison of applicator-reported and AgWeatherNet wind conditions for each drift event in all crops (n=57). Time series plots for wind speeds indicate how long each spray event lasted in terms of hours (some lasted longer than 8 hours). Applicator-Low, -Avg, and -High show range of wind speeds reported on the corresponding application record. Top grey panel denotes event year and right grey panel denotes distance in miles between the event location and the nearest AgWeatherNet station. Wind roses constructed from AgWeatherNet data indicate direction from which the wind was blowing, wind speed category, and percent of time that the wind blew from each direction category during the spray event. Wind direction category listed on the far right was from the application record (note: [forms did not indicate whether wind category was “to” or “from” that particular direction](https://agr.wa.gov/PestFert/Pesticides/docs/RecForm4226.pdf)).

| **Wind Speed** |  | **Wind Direction** | | |
| --- | --- | --- | --- | --- |
|  |  | **AgWeatherNet** |  | **Applicator** |


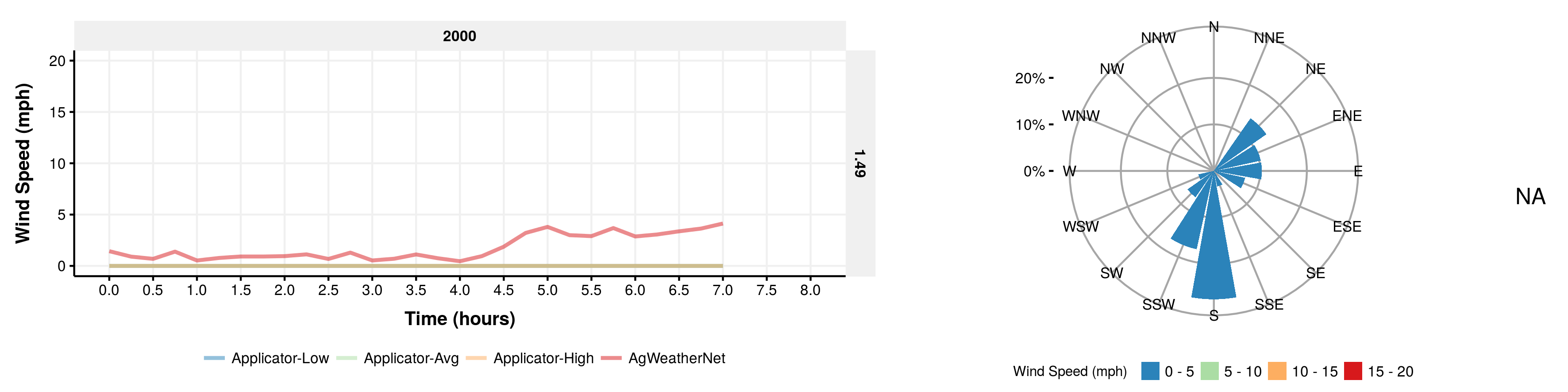

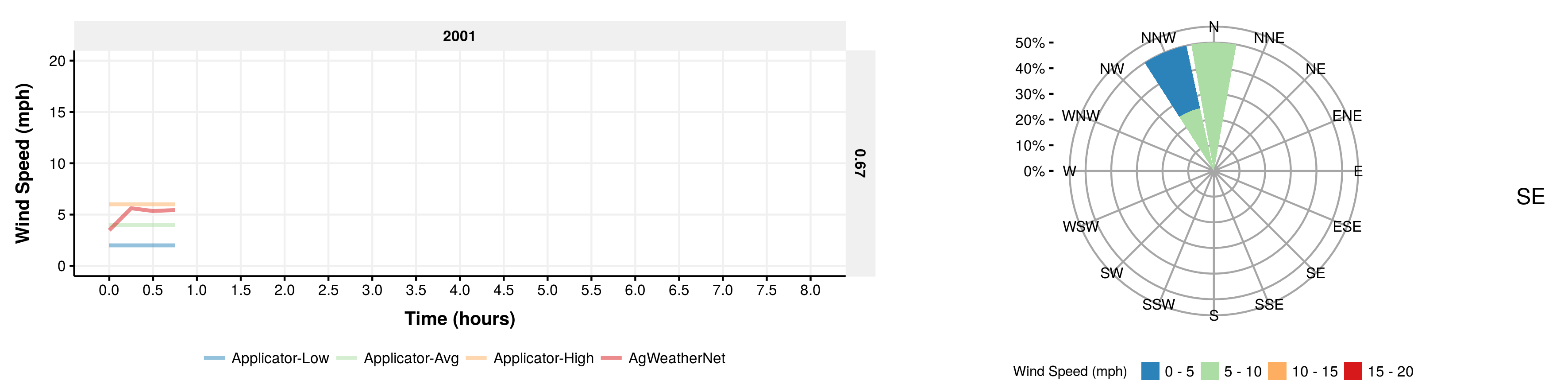

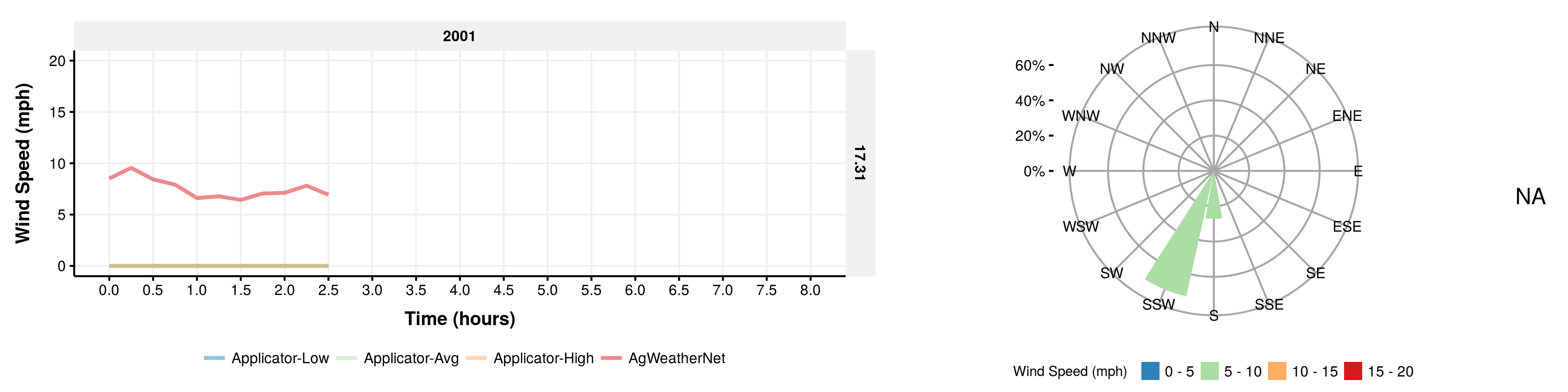

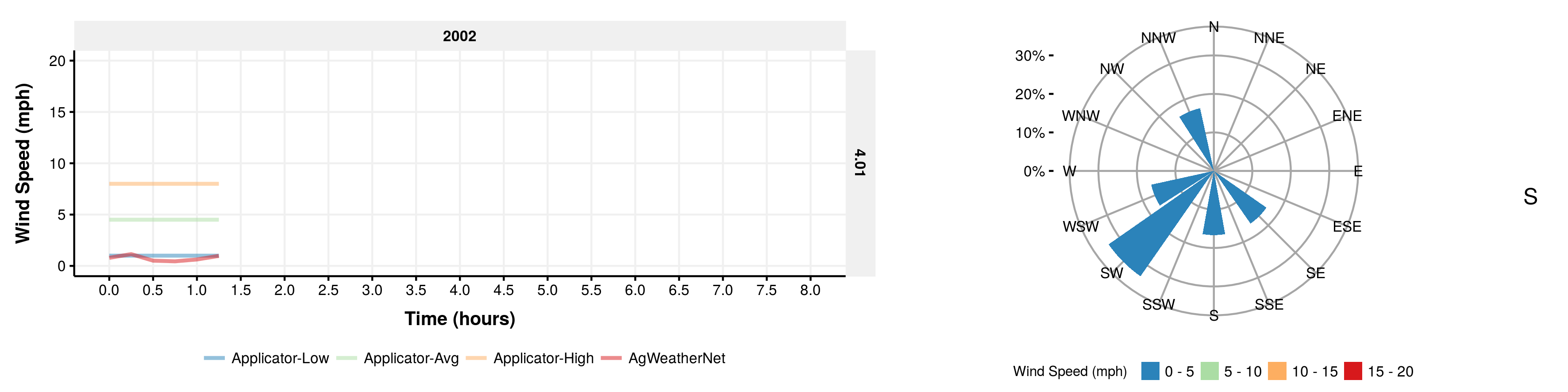

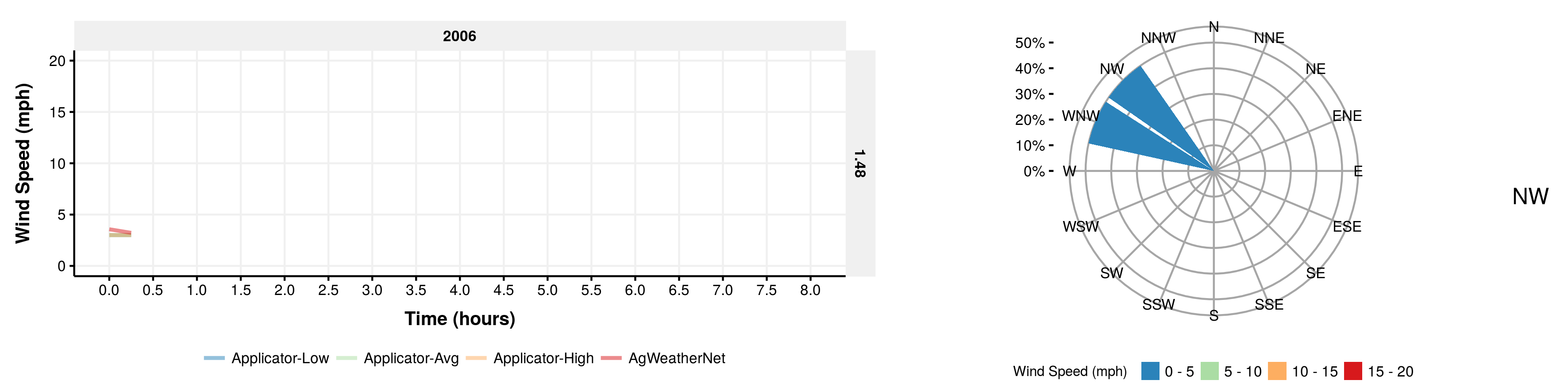

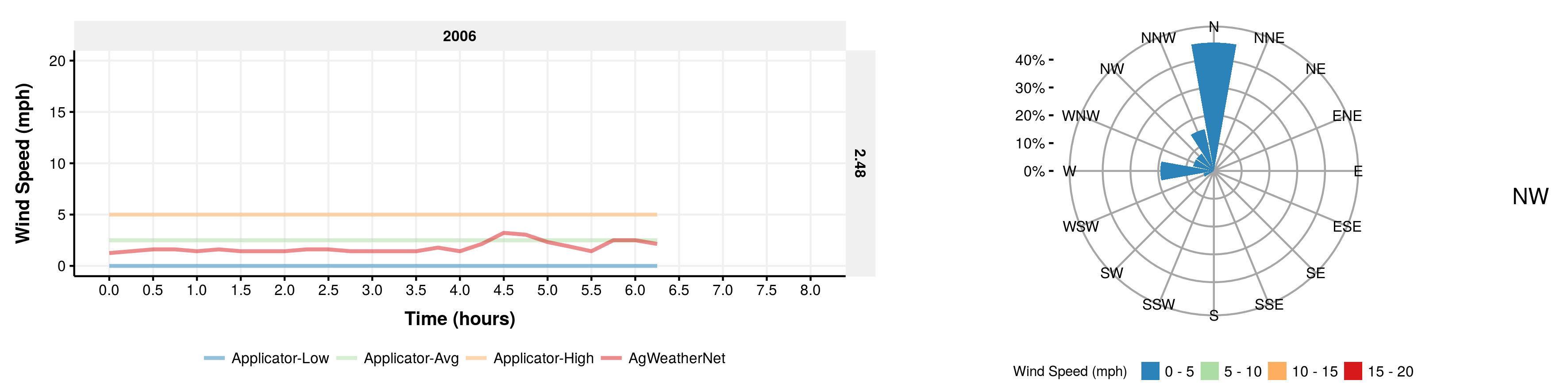

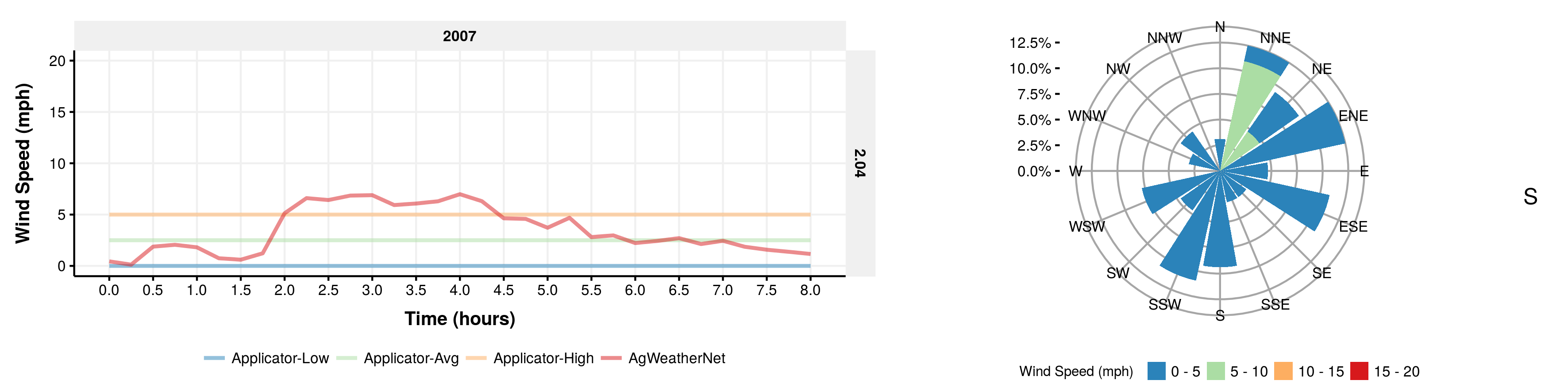

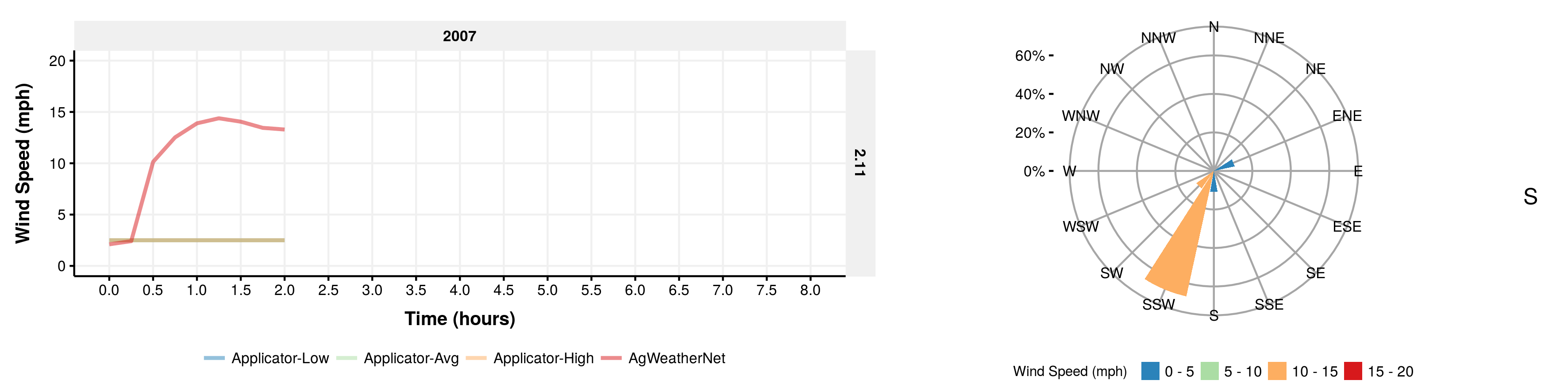

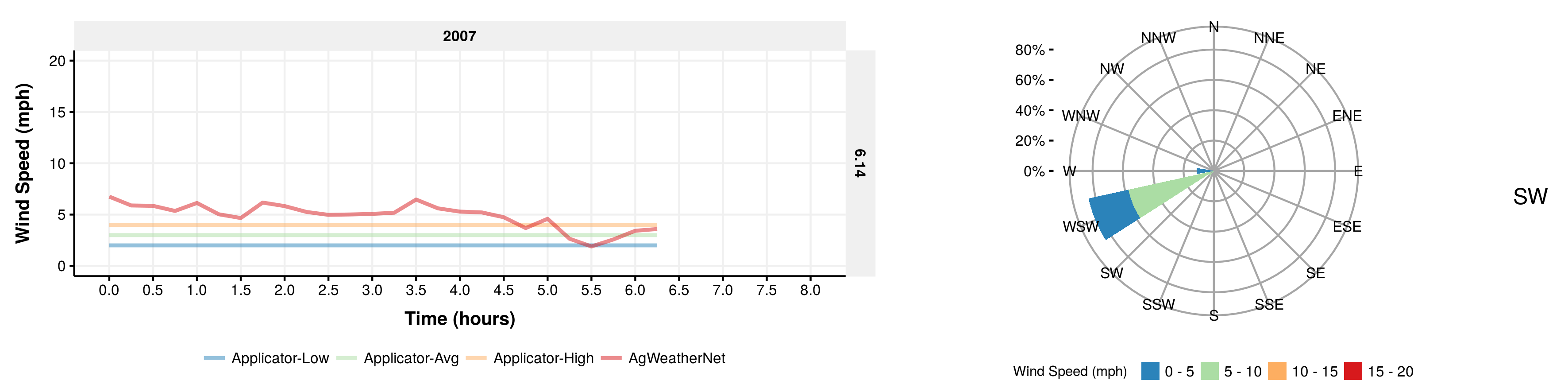

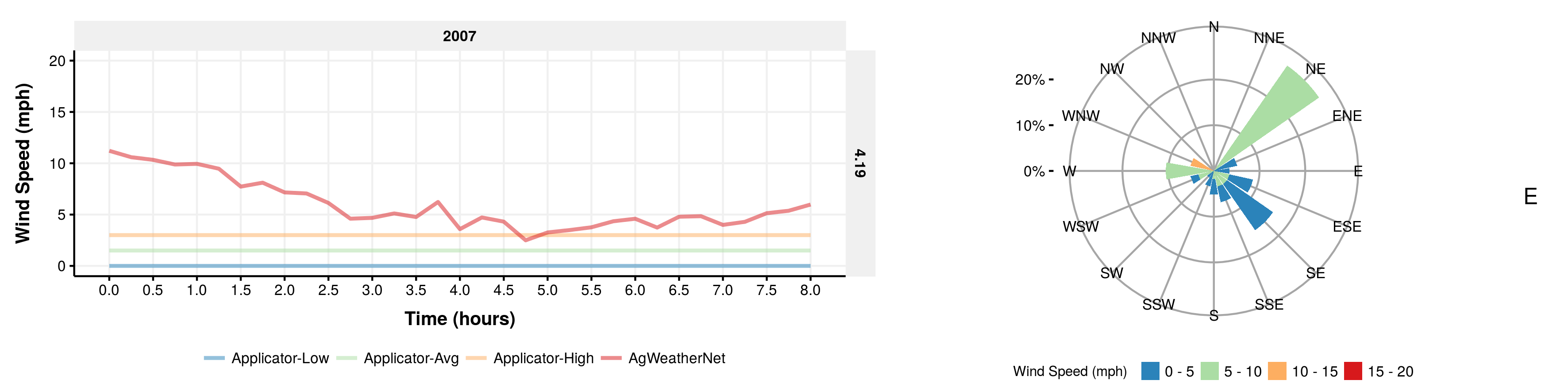

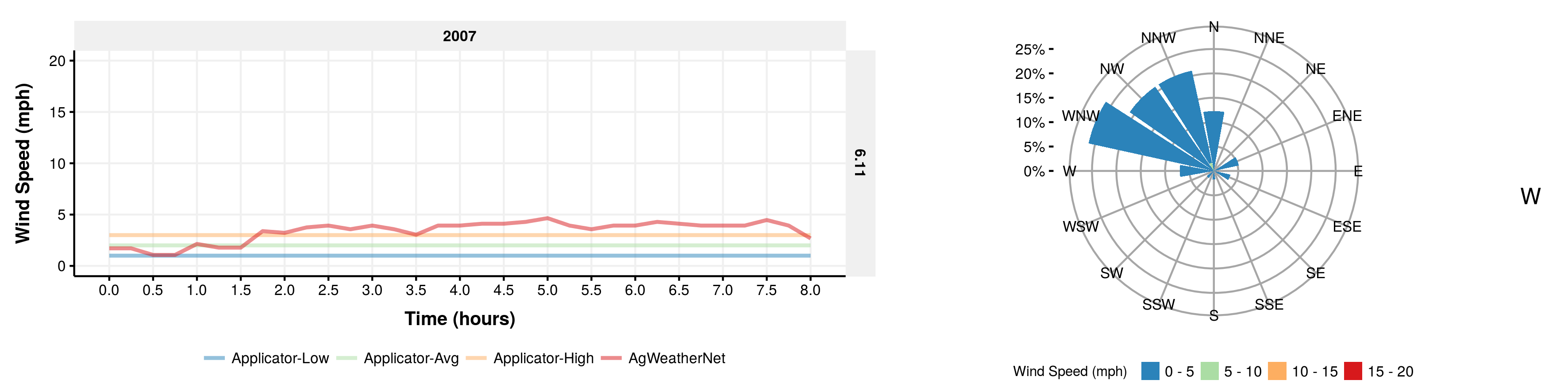

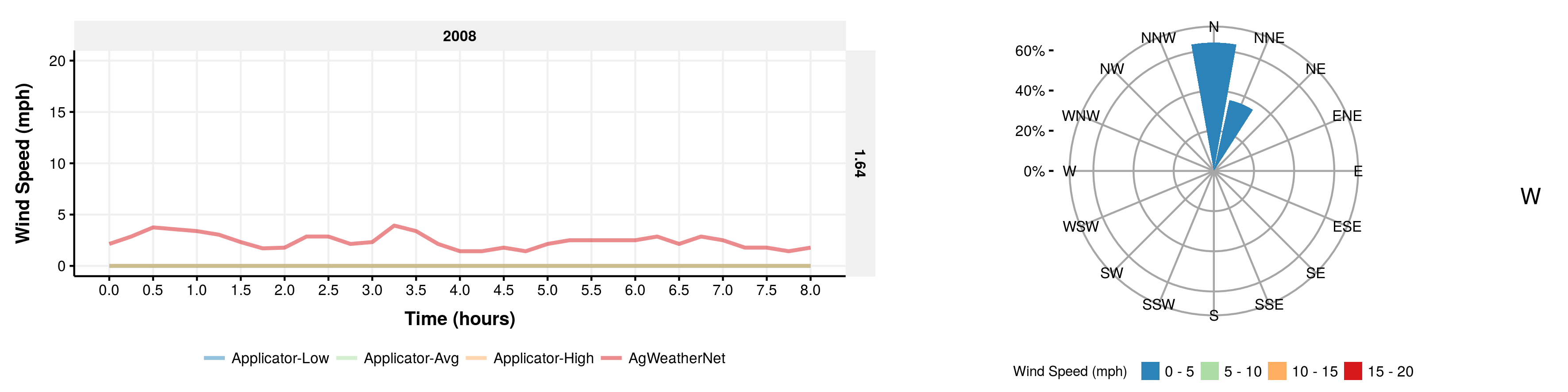

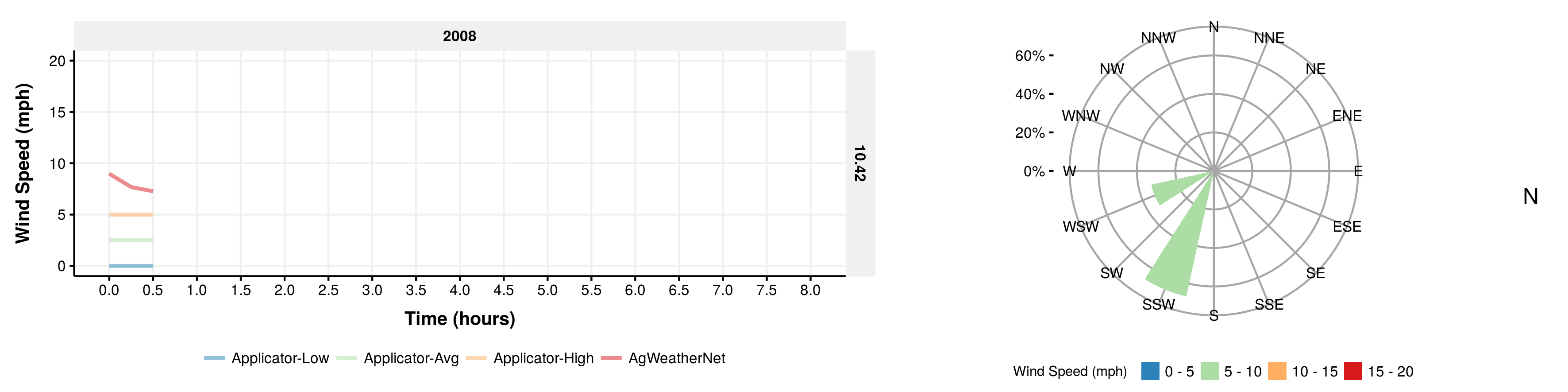

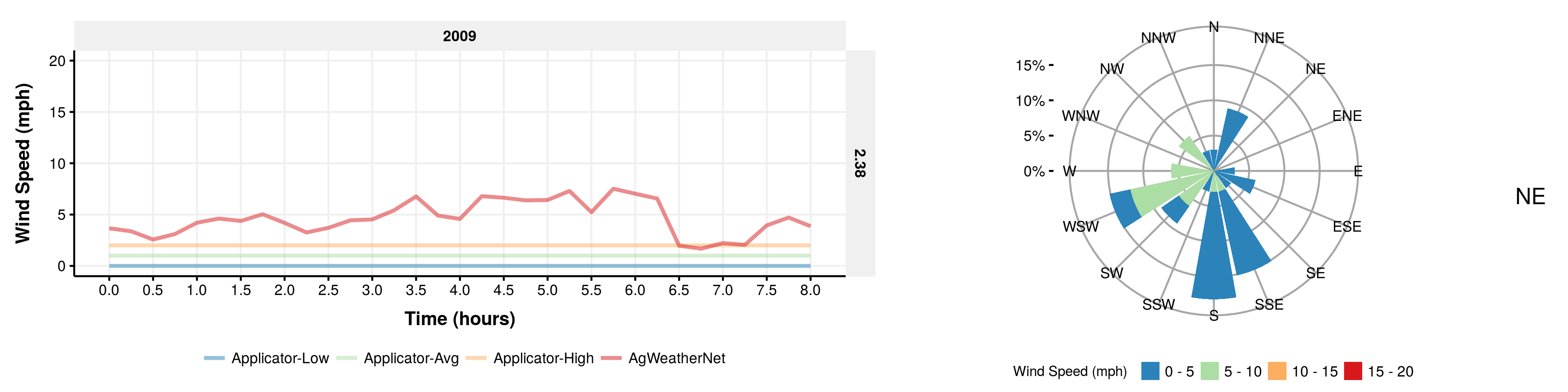

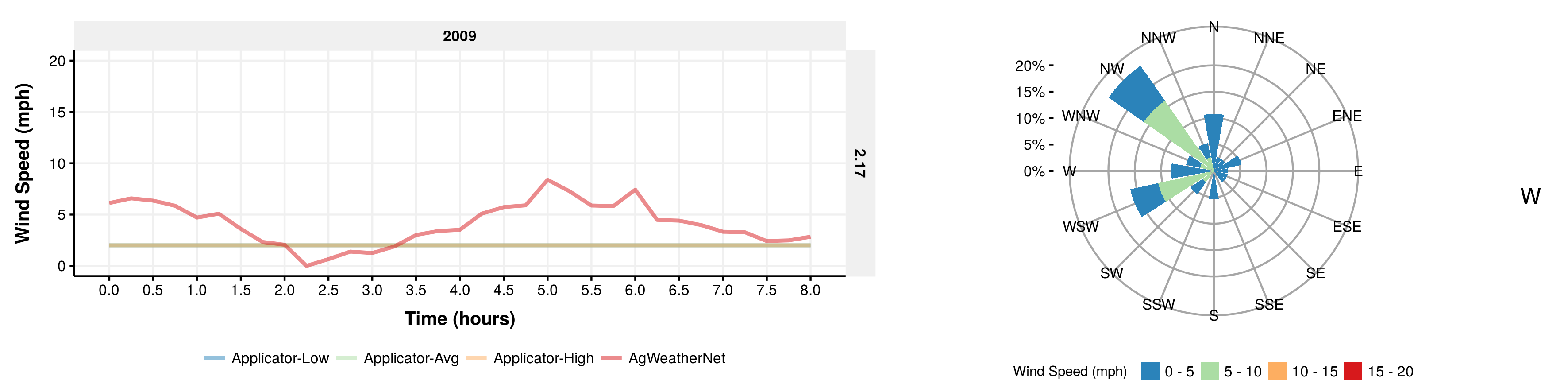

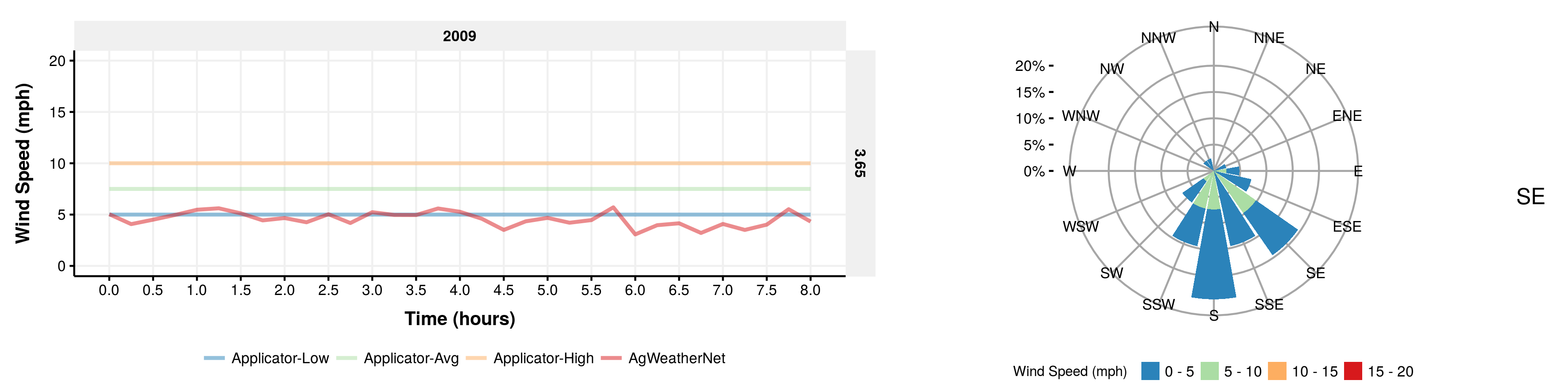

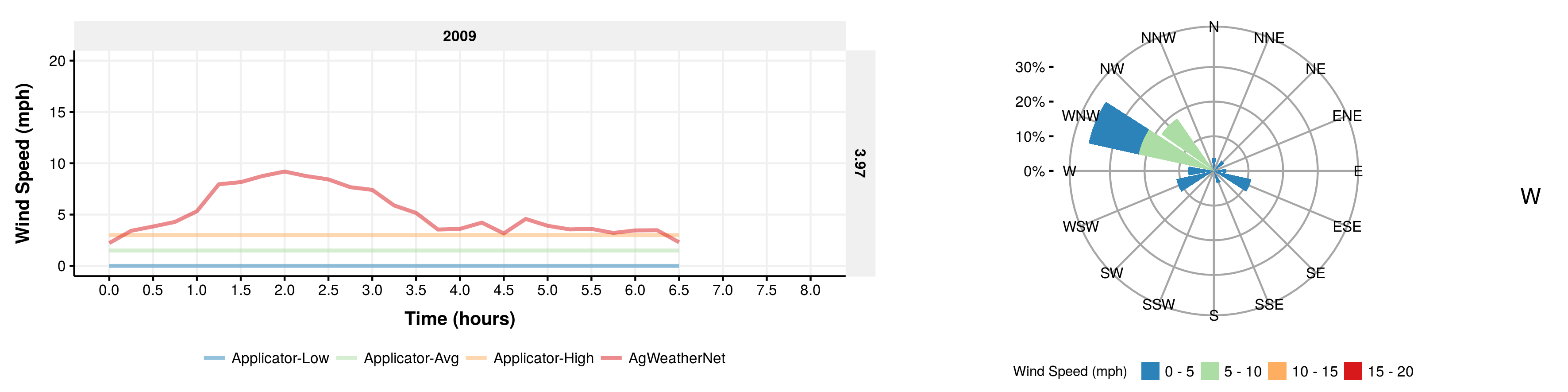

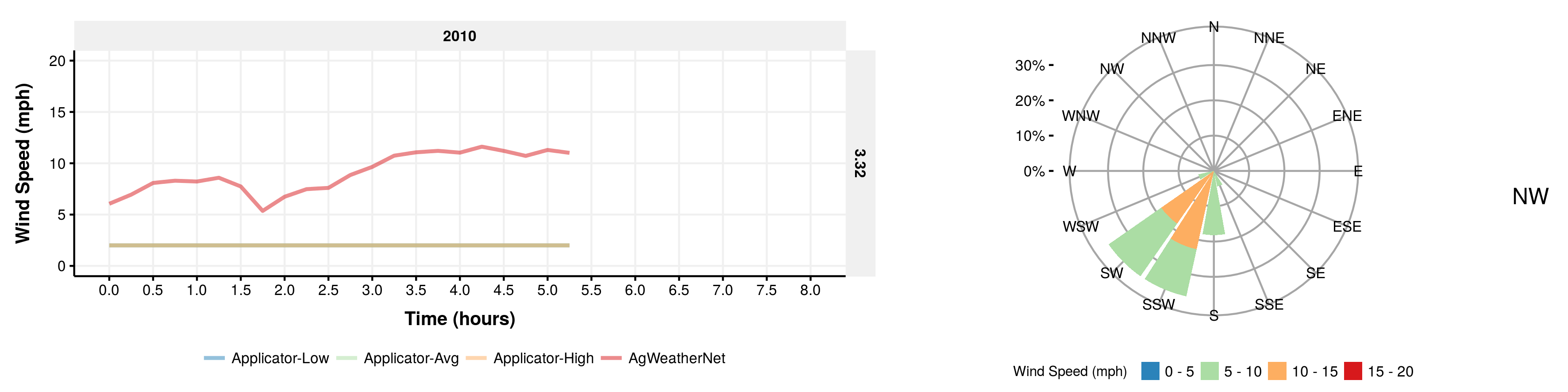

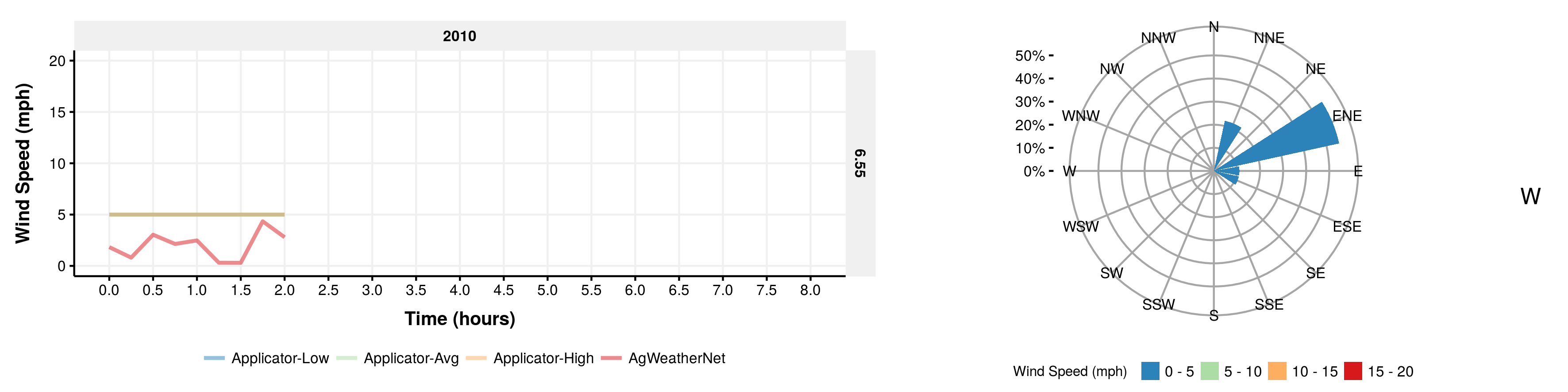

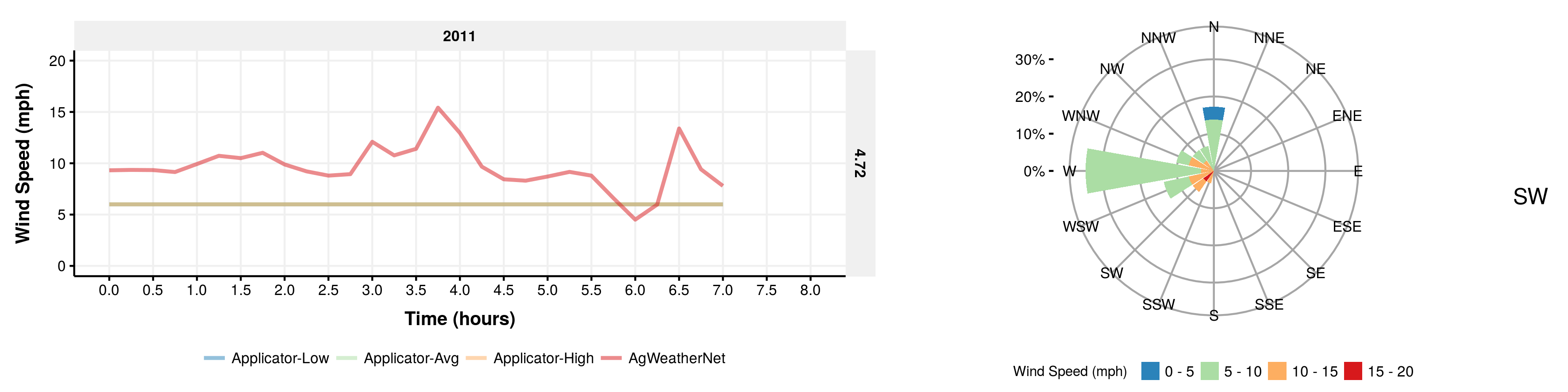

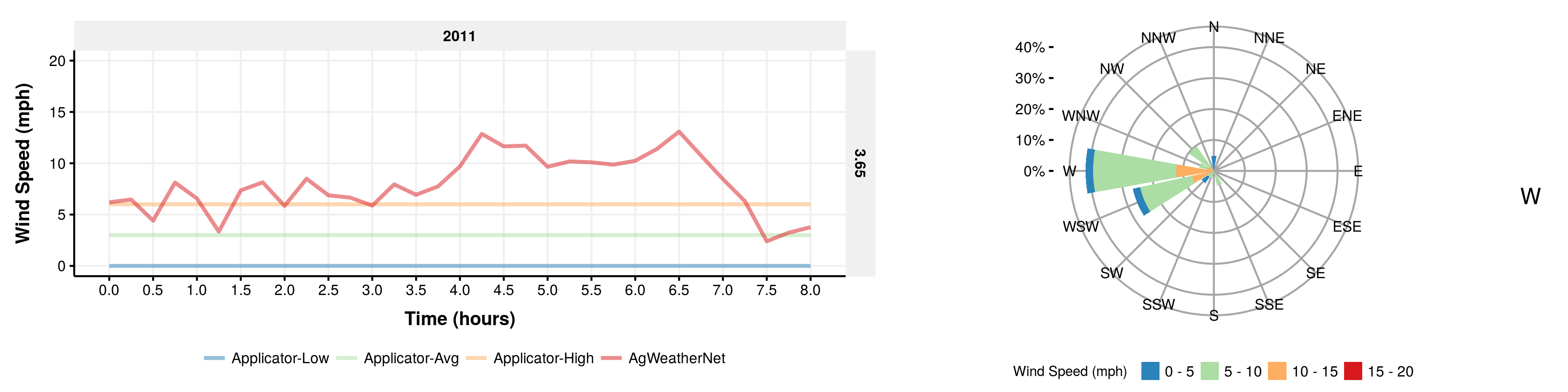

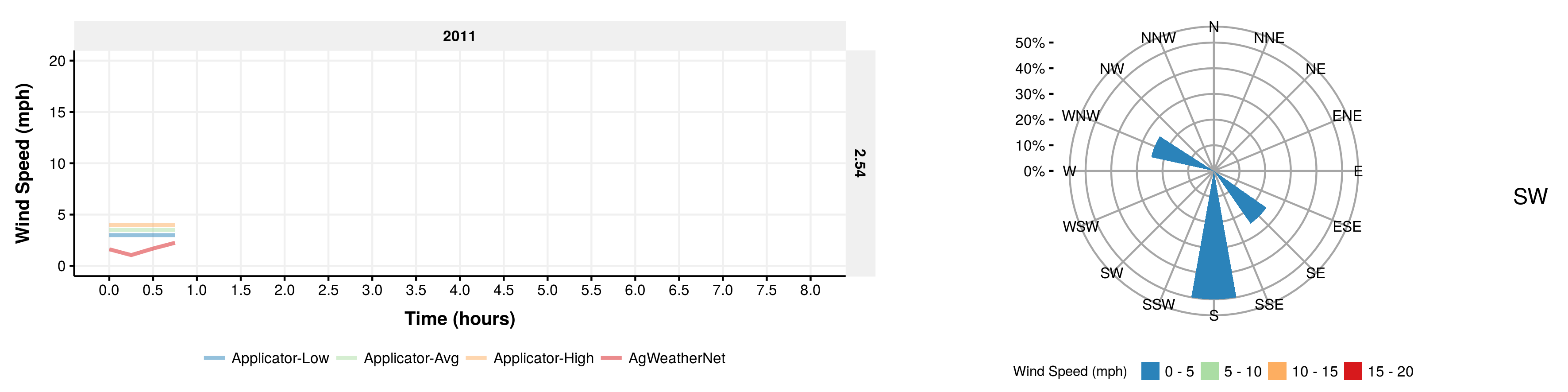

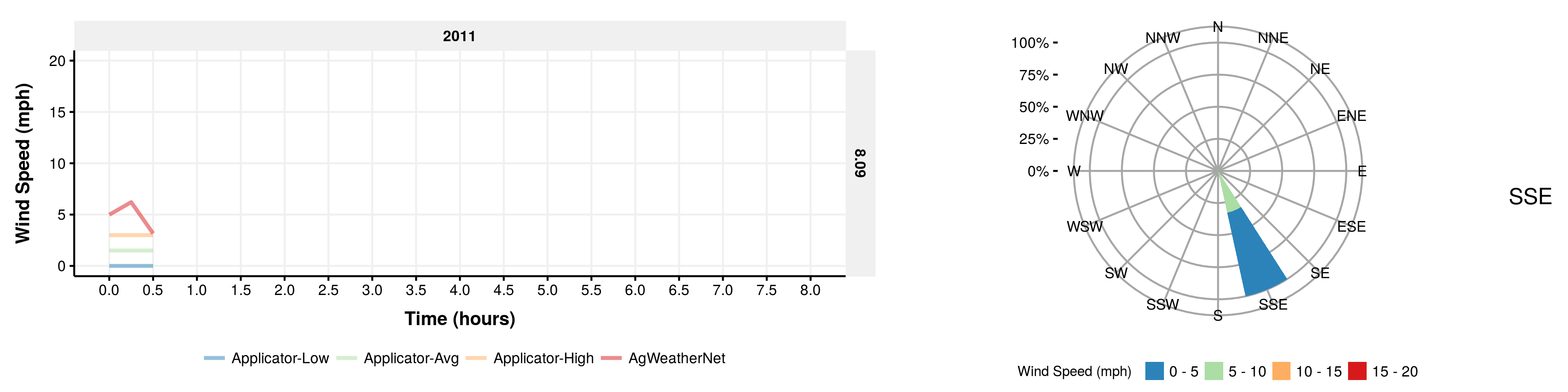

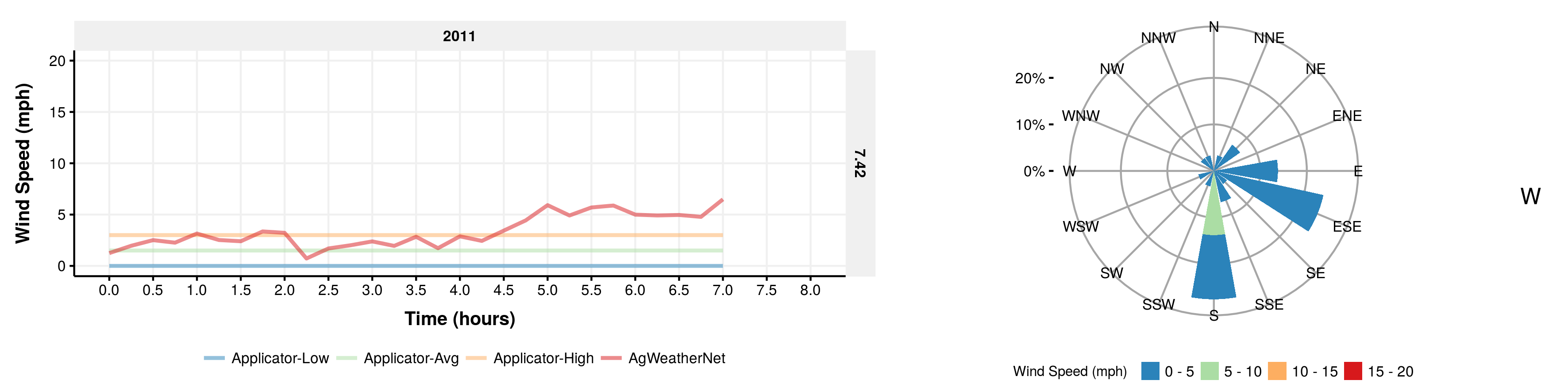

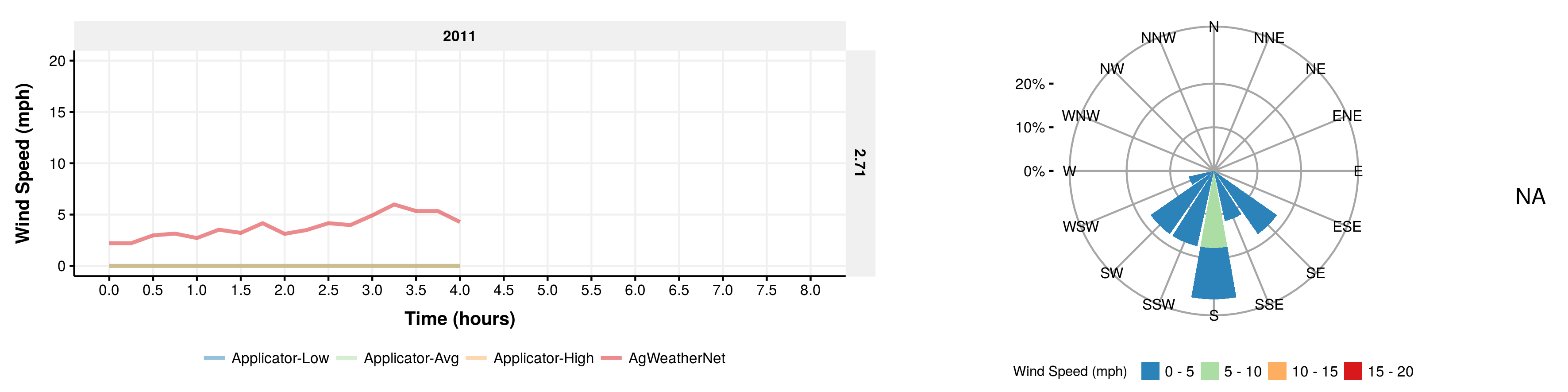

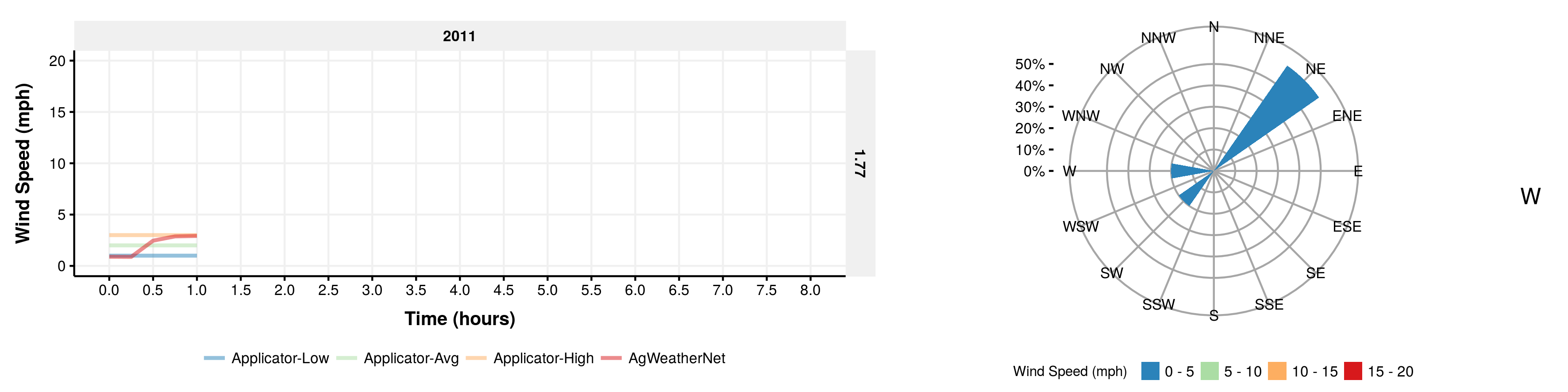

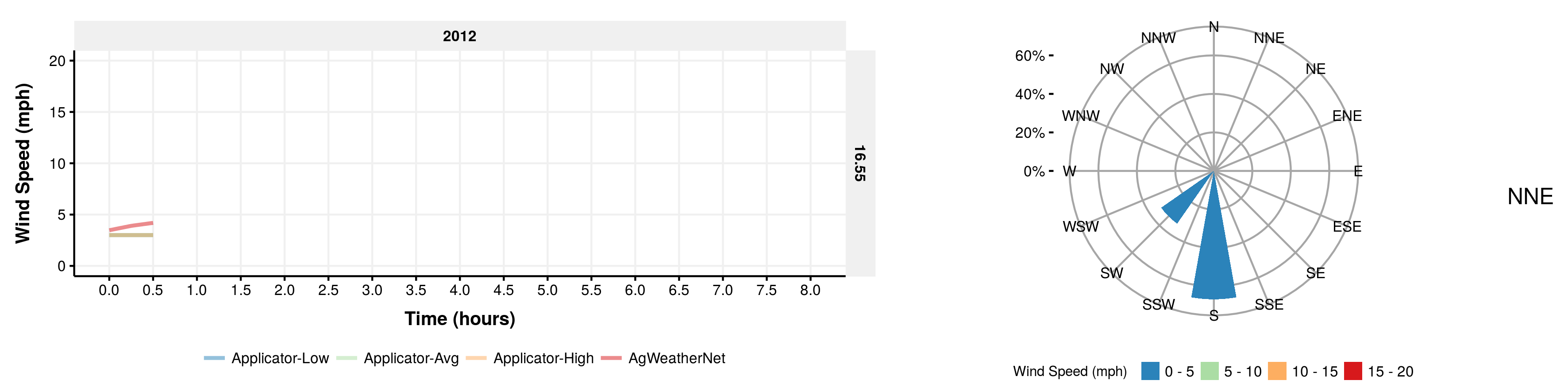

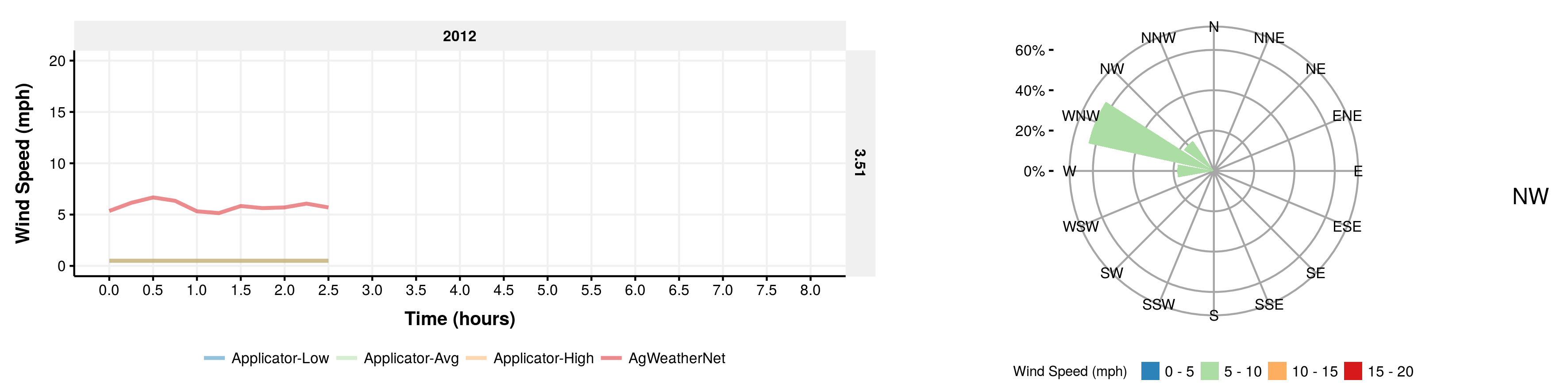

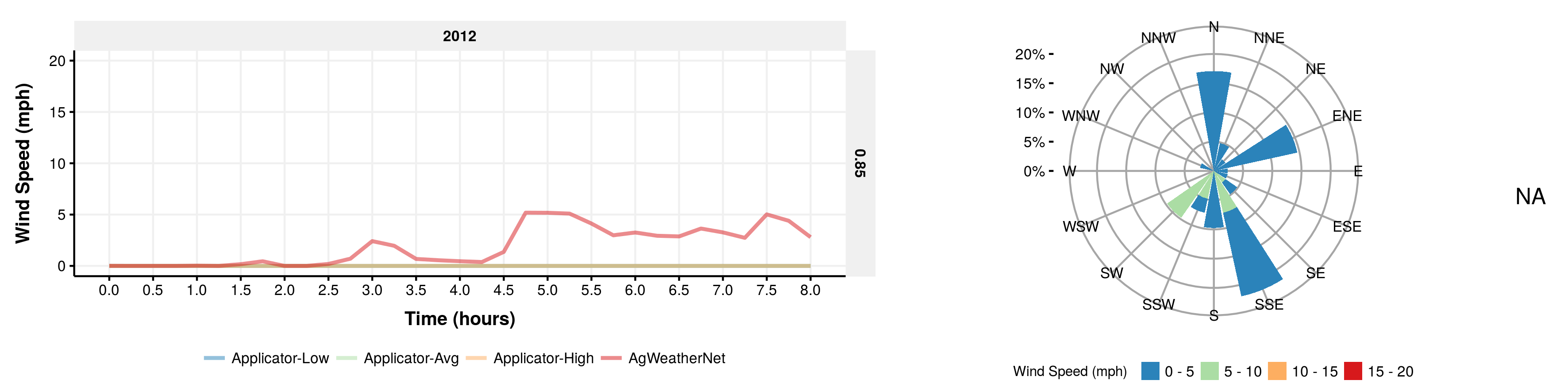

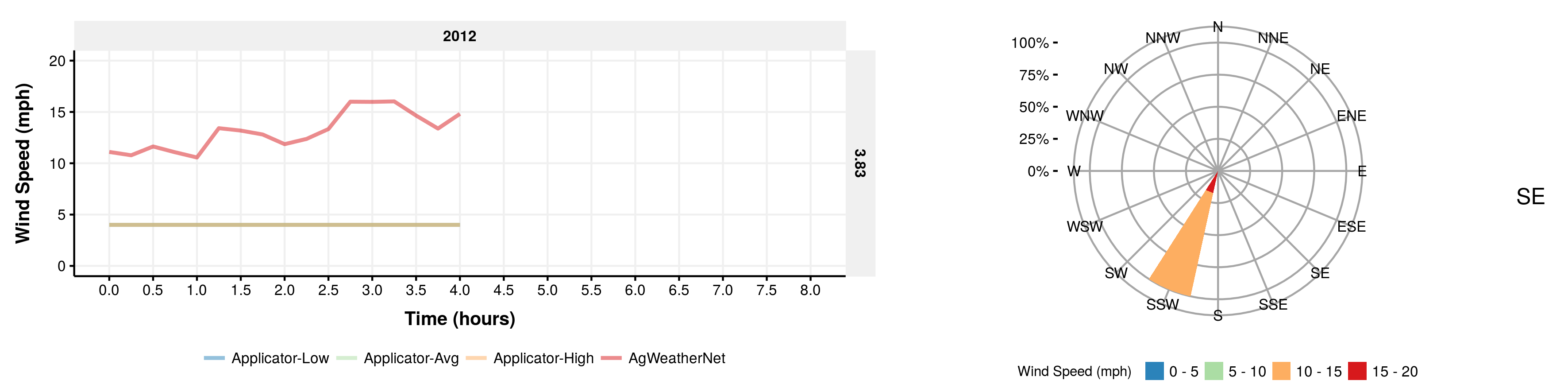

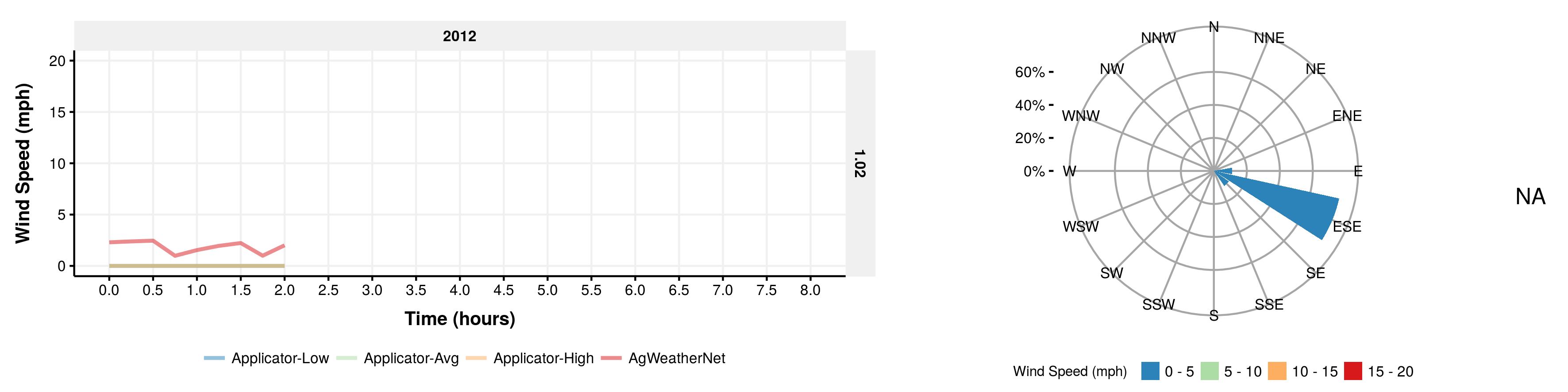

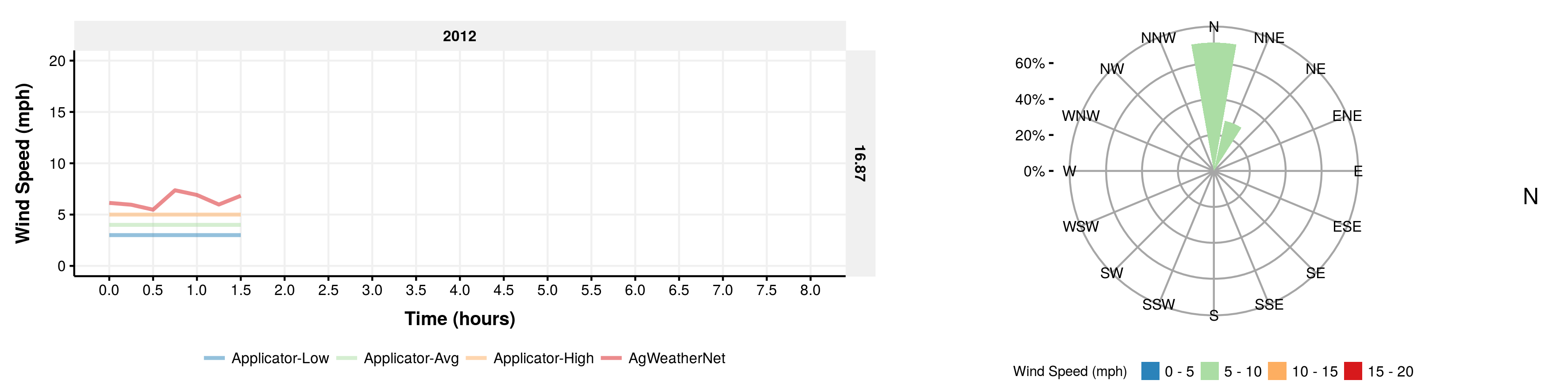

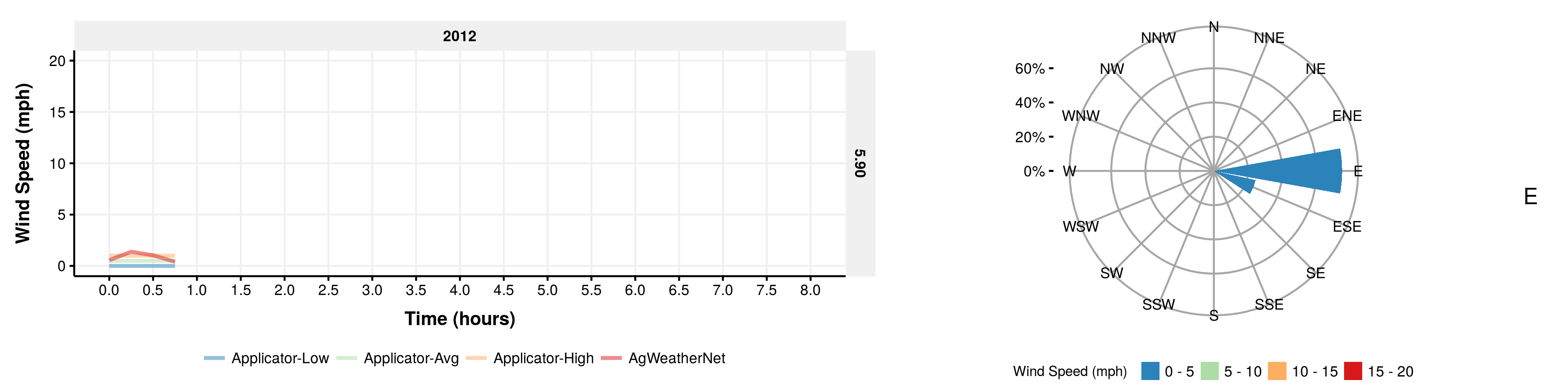

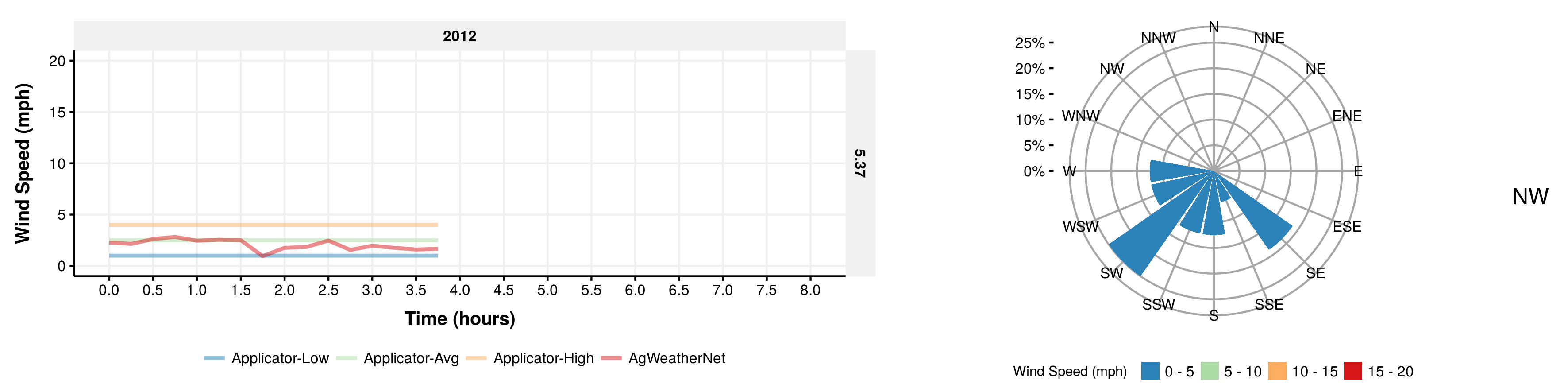

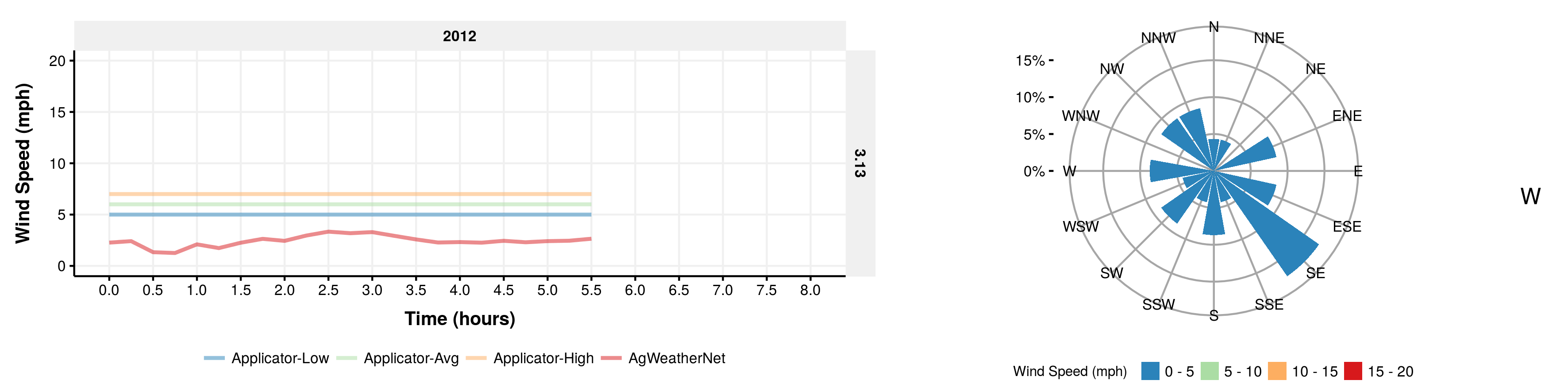

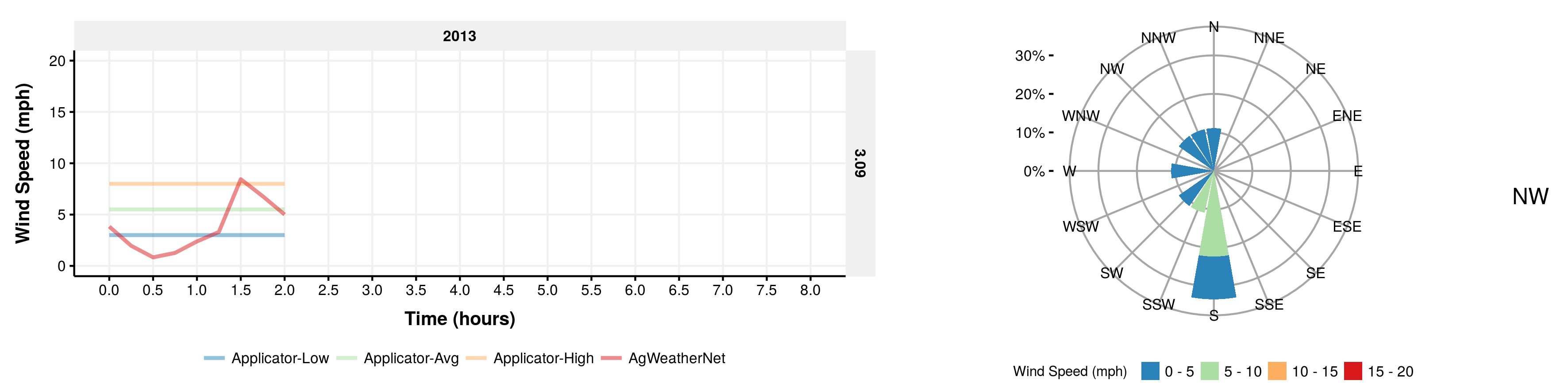

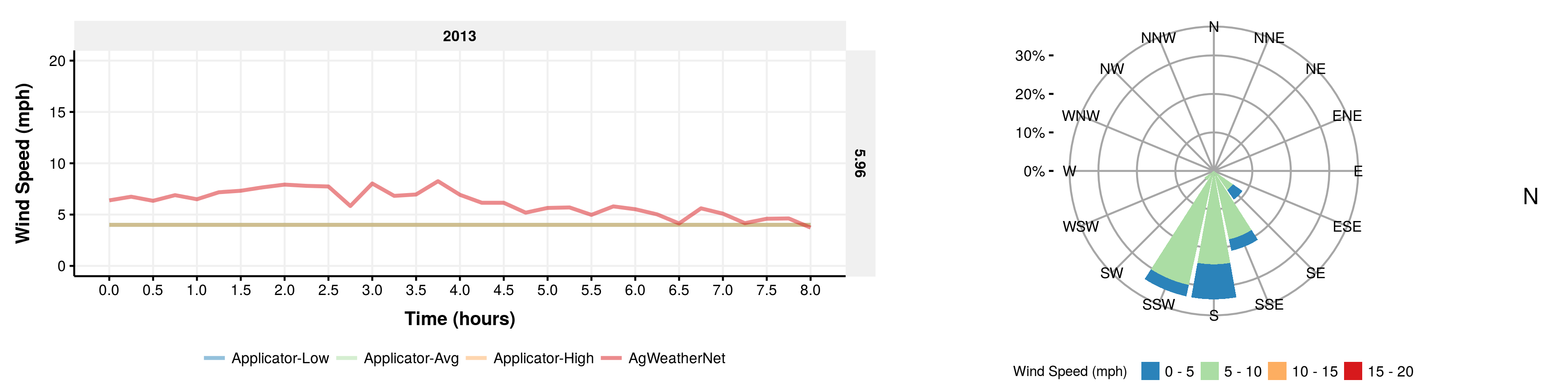

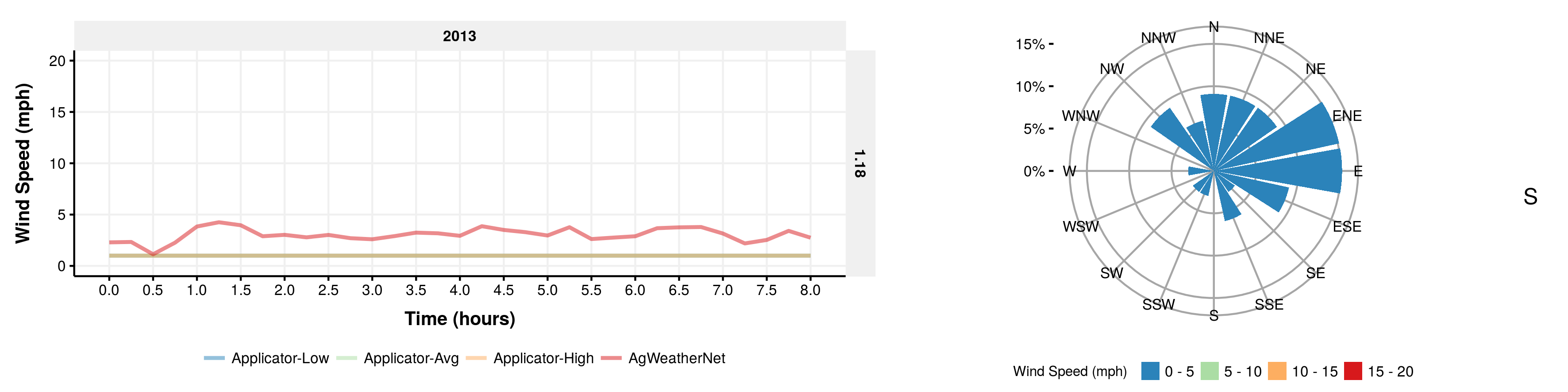

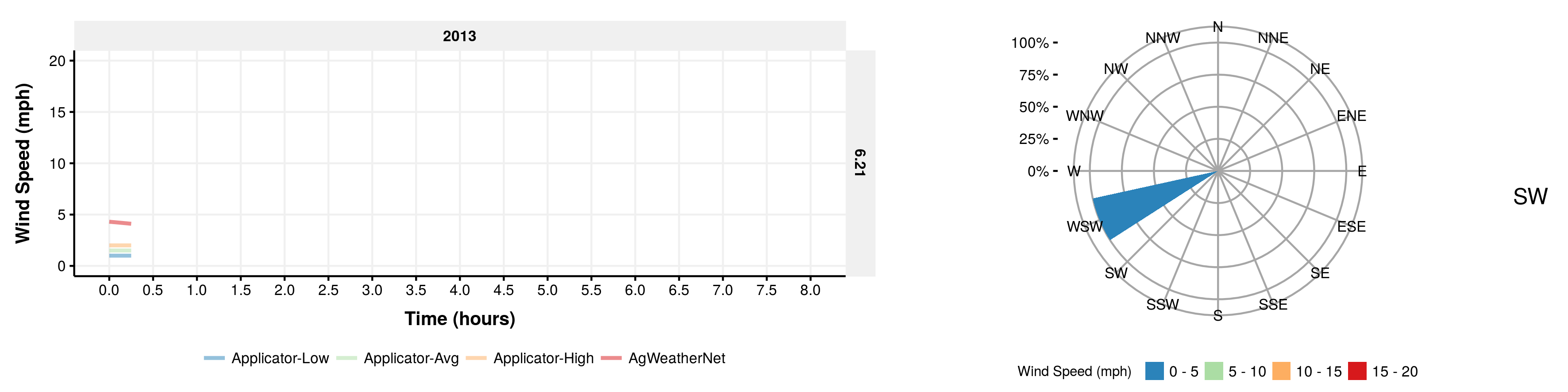

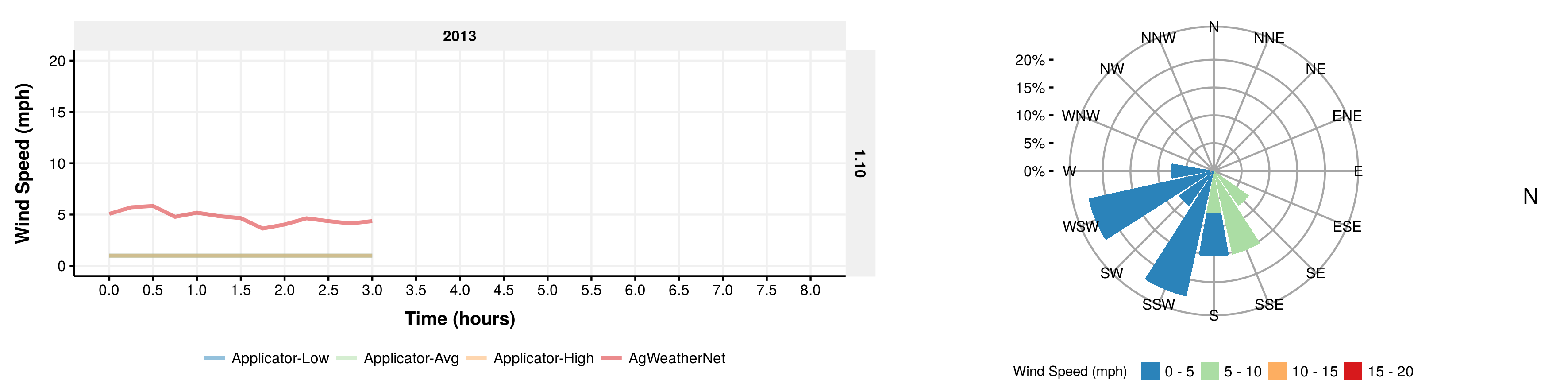

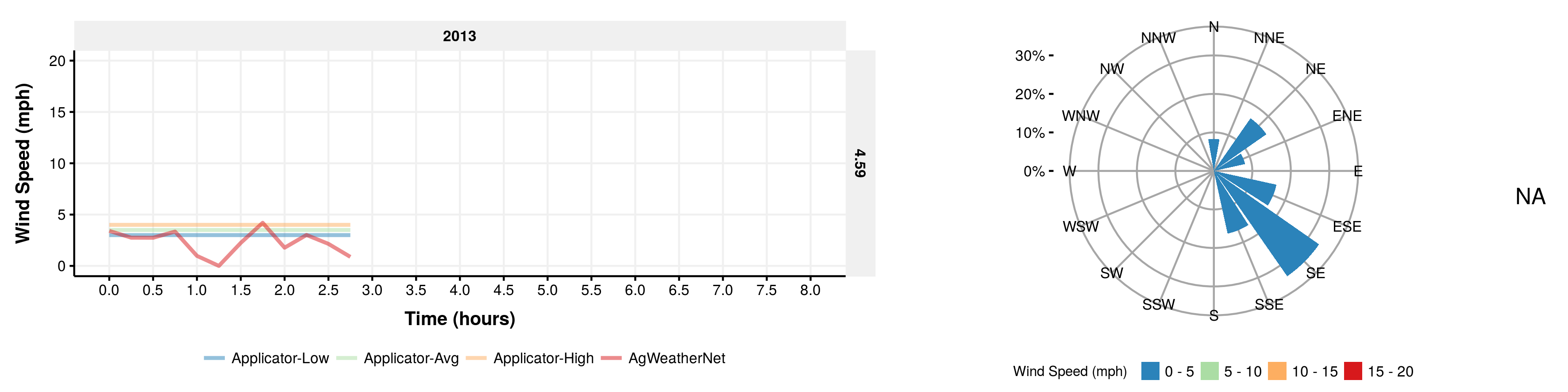

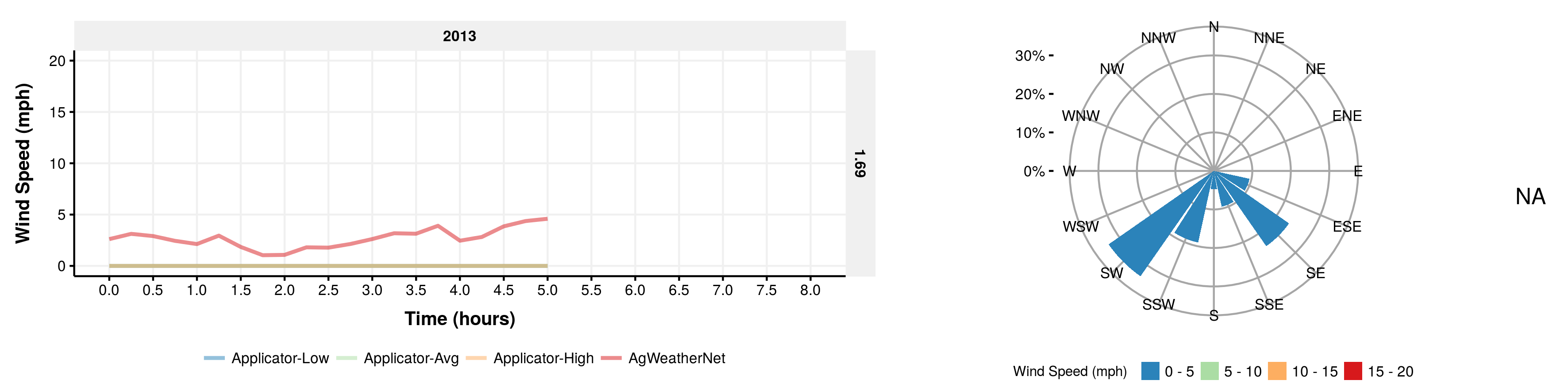

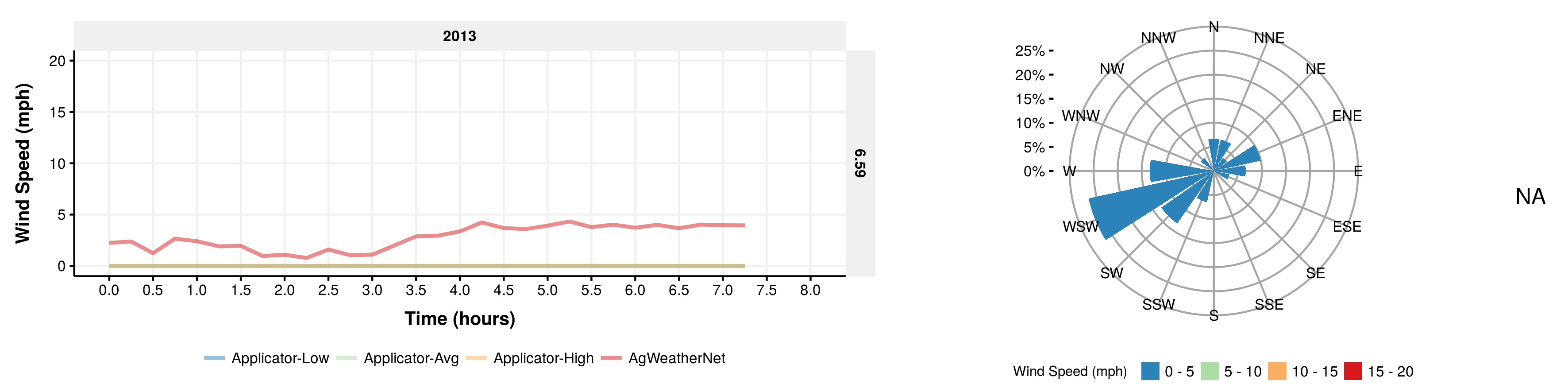

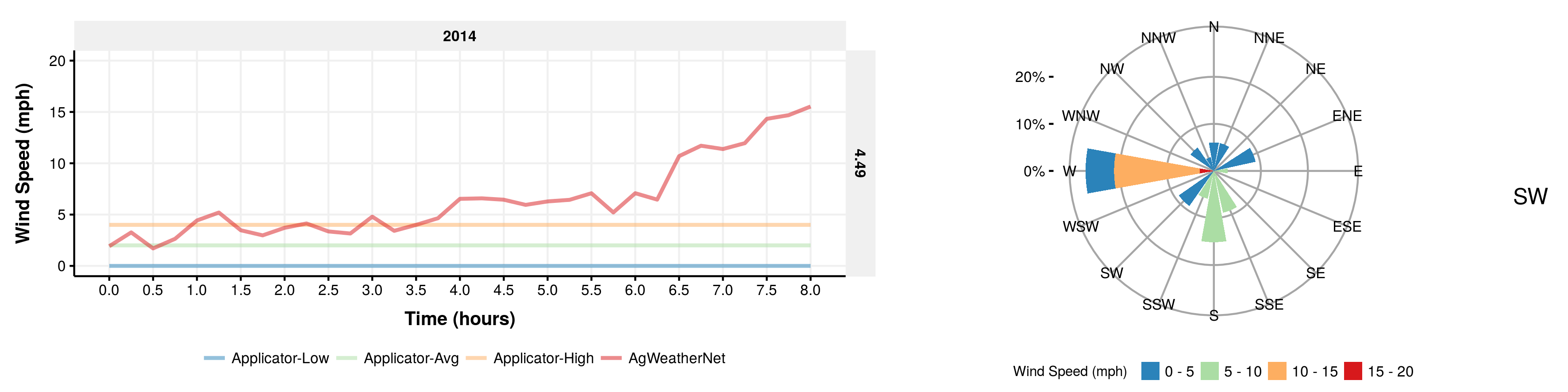

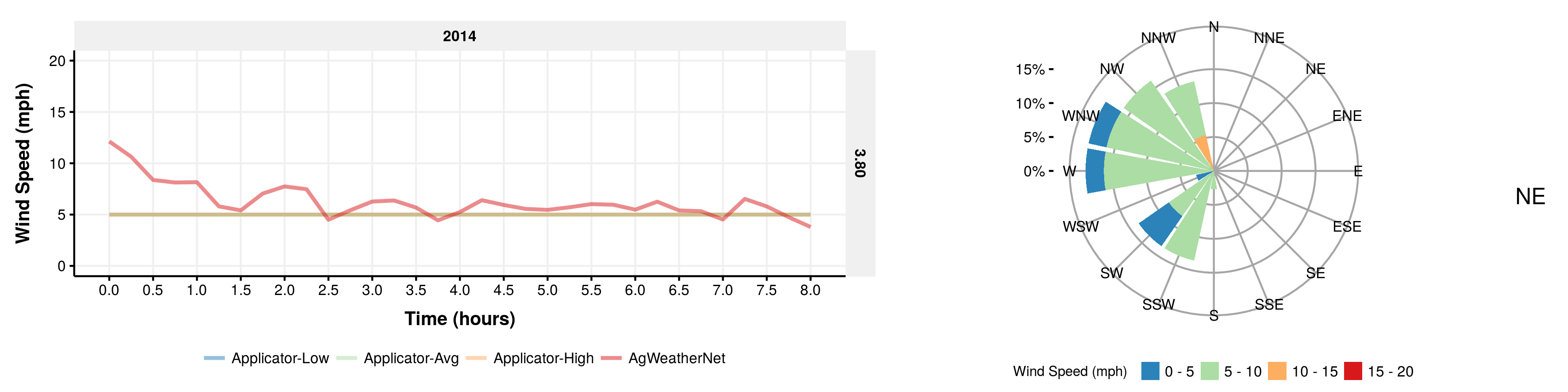

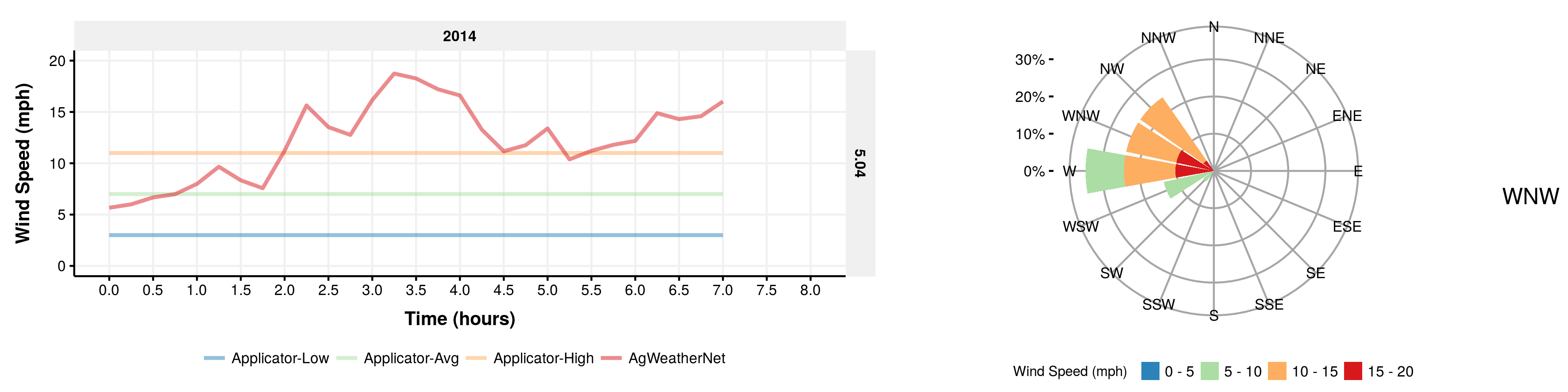

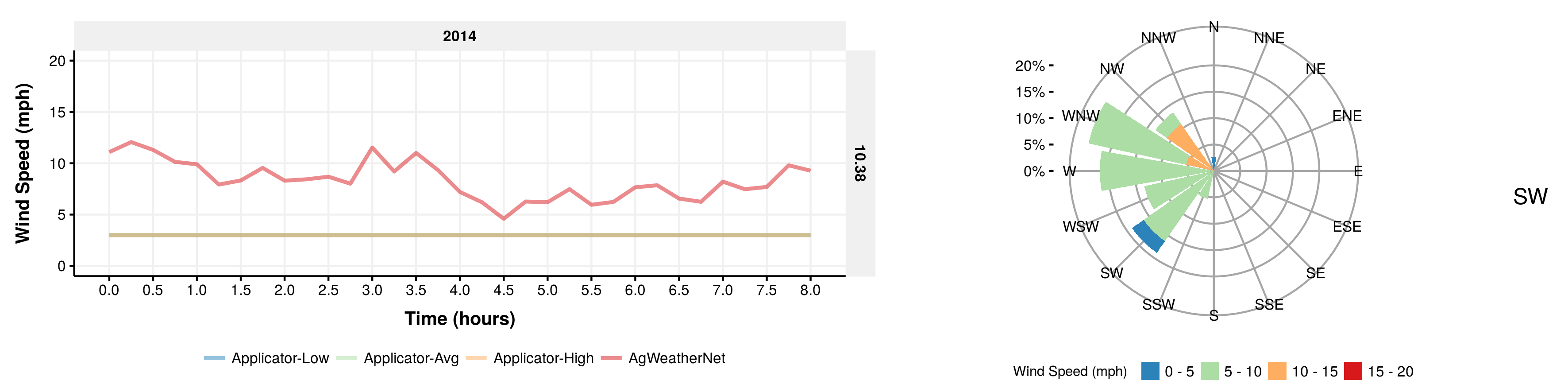

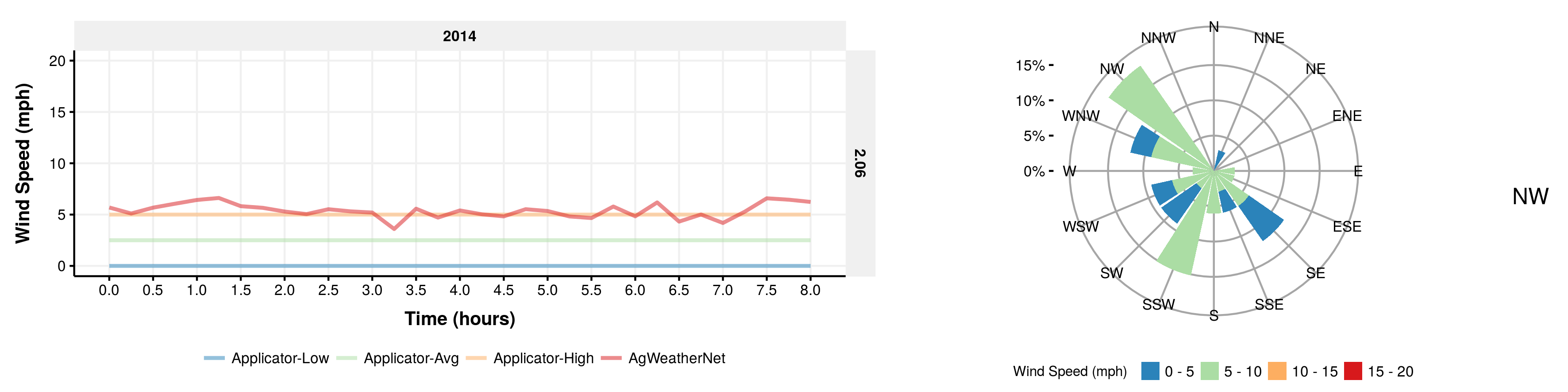

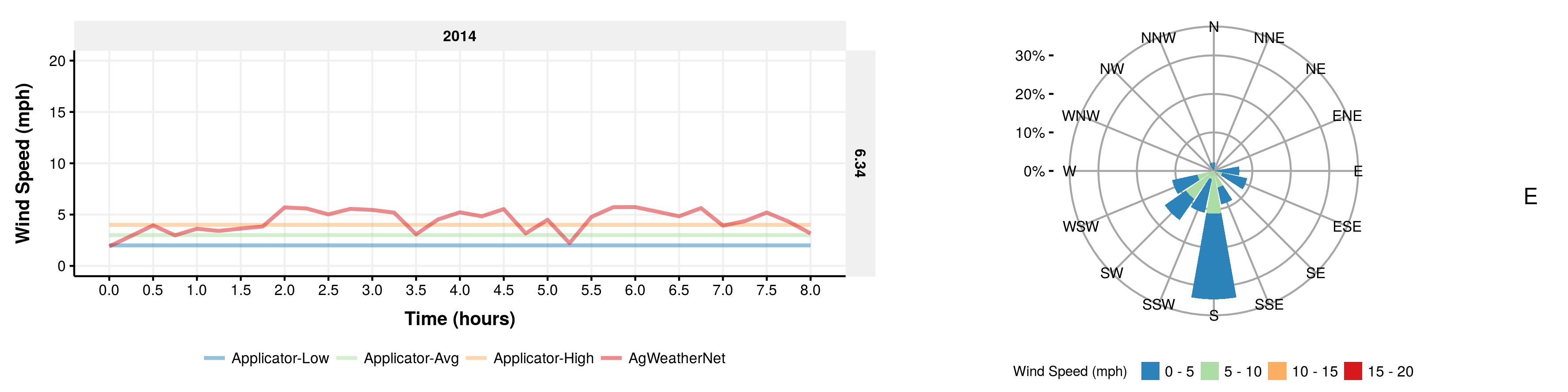

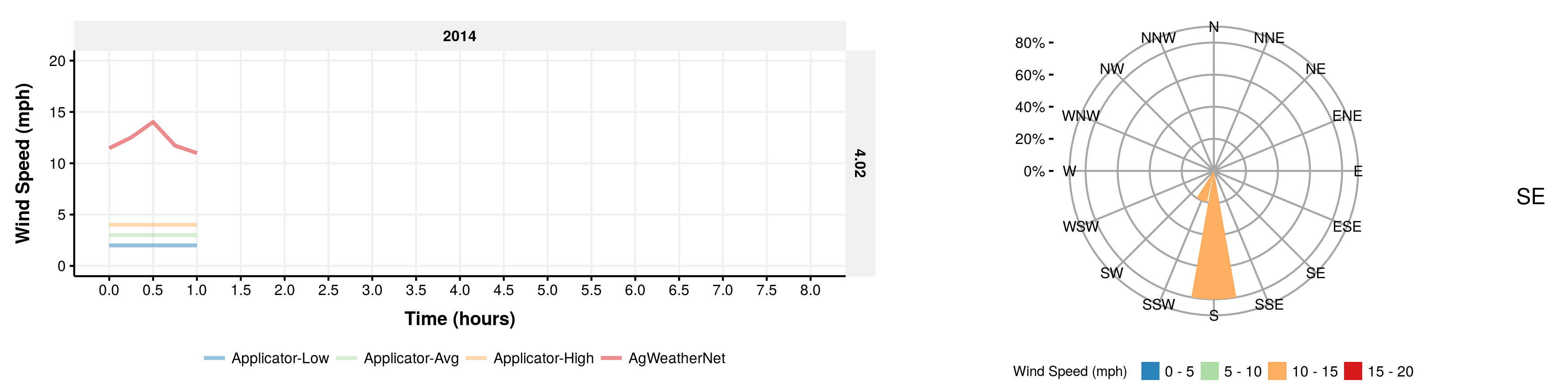

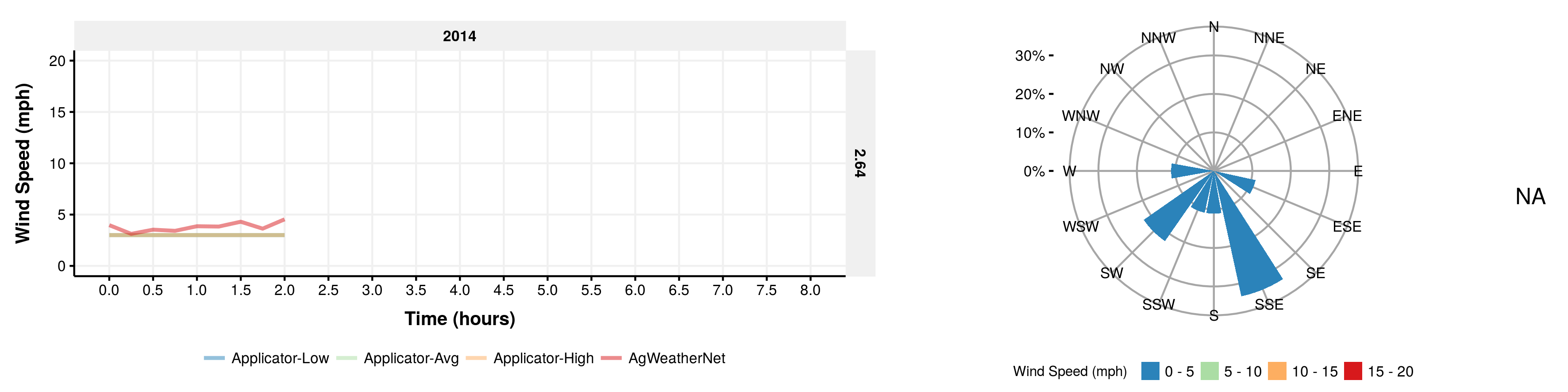

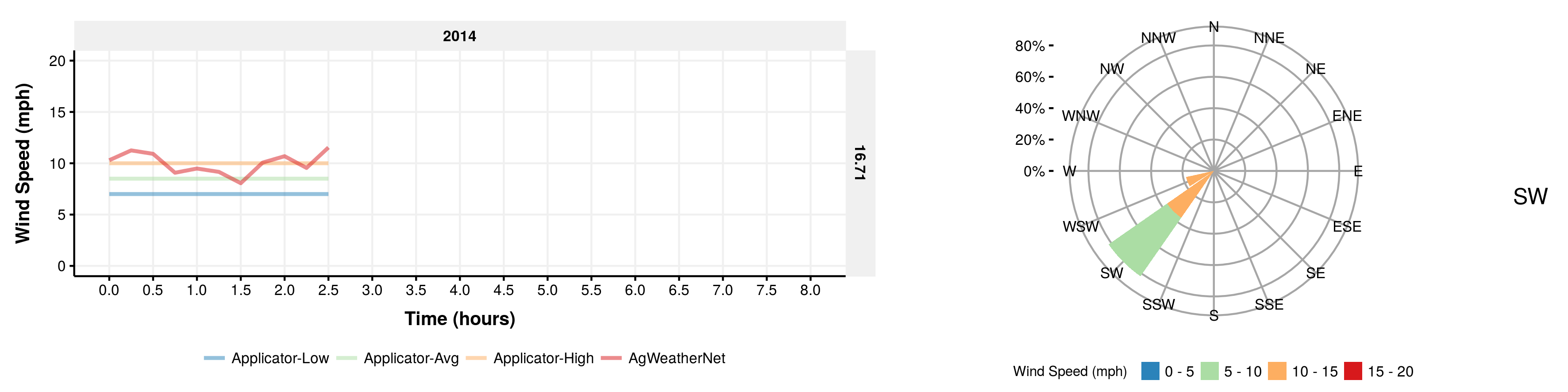

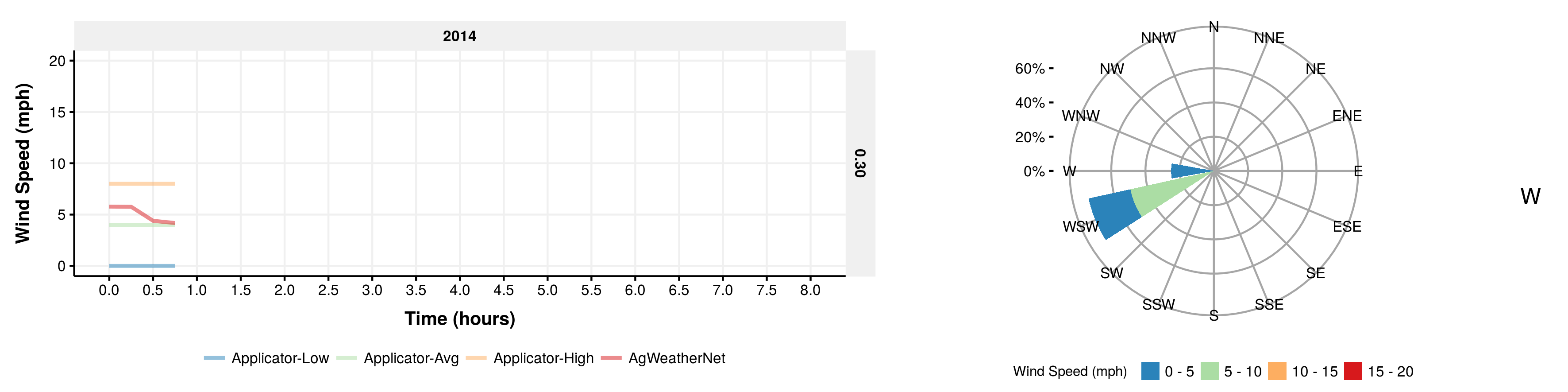

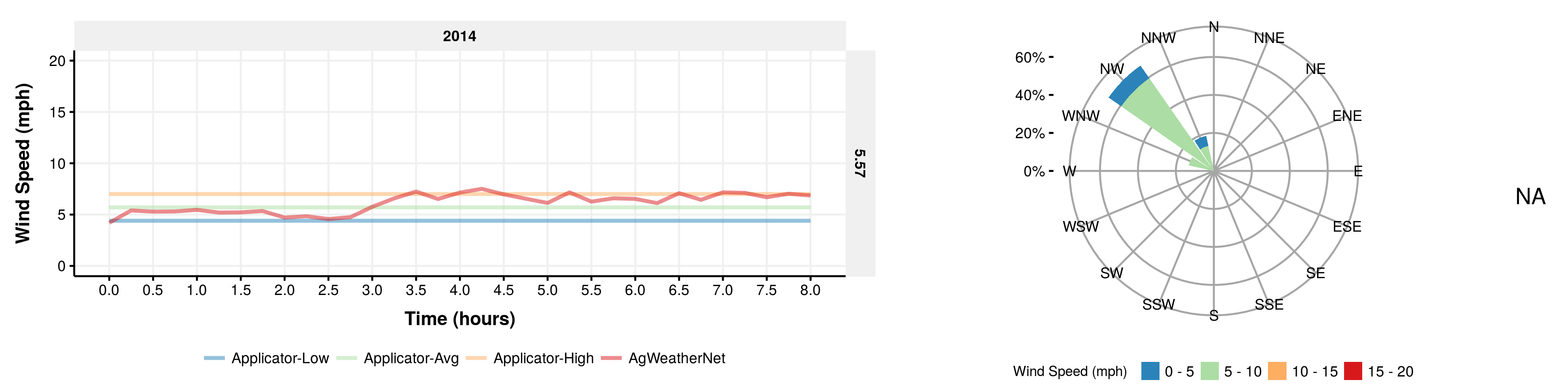

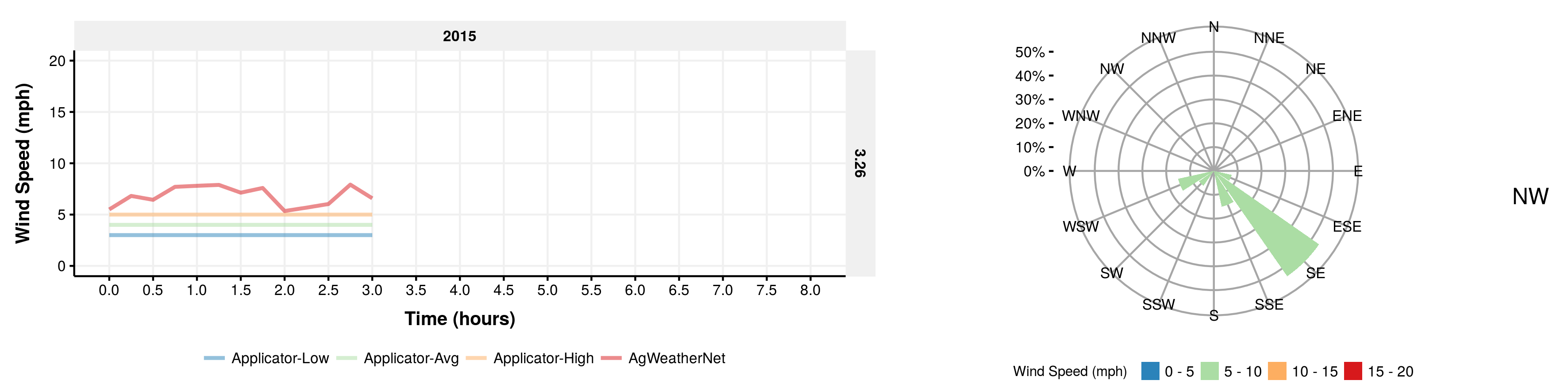

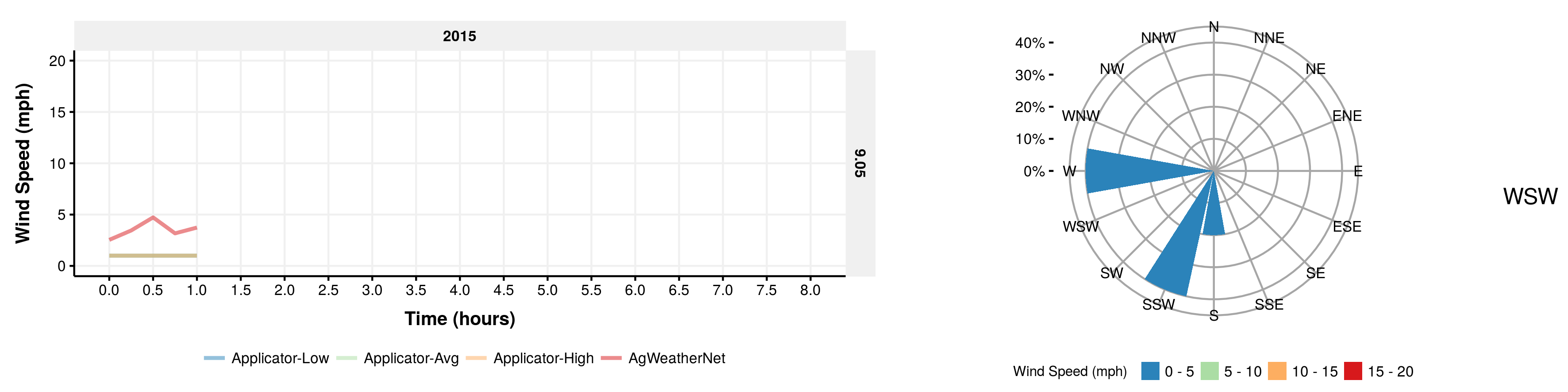

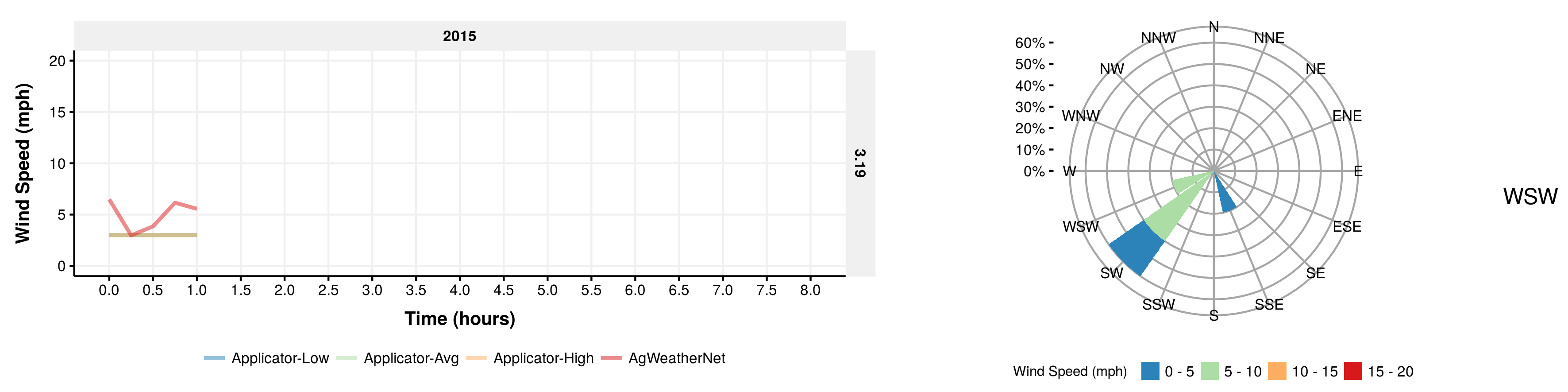

Supplement: Supplementary file 1 — Additional file 1. [file 12940_2021_693_MOESM1_ESM.docx]
